# Multi-omics of Circular RNAs and Their Responses to Hormones in Moso Bamboo (*Phyllostachys edulis*)

**DOI:** 10.1016/j.gpb.2023.01.007

**Published:** 2023-02-16

**Authors:** Yongsheng Wang, Huihui Wang, Huiyuan Wang, Ruifan Zhou, Ji Wu, Zekun Zhang, Yandong Jin, Tao Li, Markus V. Kohnen, Xuqing Liu, Wentao Wei, Kai Chen, Yubang Gao, Jiazhi Ding, Hangxiao Zhang, Bo Liu, Chentao Lin, Lianfeng Gu

**Affiliations:** 1 Basic Forestry and Proteomics Research Center, College of Forestry, Fujian Provincial Key Laboratory of Haixia Applied Plant Systems Biology, Fujian Agriculture and Forestry University, Fuzhou 350002, China; 2 College of Forestry, Fujian Agriculture and Forestry University, Fuzhou 350002, China; 3 Department of Molecular, Cell and Developmental Biology, University of California at Los Angeles, Los Angeles, CA 90095, USA

**Keywords:** Alternative splicing, Circular RNA, Degradome, *Phyllostachys edulis*, Phytohormone

## Abstract

**Circular RNAs** (circRNAs) are endogenous non-coding RNAs with covalently closed structures, which have important functions in plants. However, their biogenesis, degradation, and function upon treatment with gibberellins (GAs) and auxins (1-naphthaleneacetic acid, NAA) remain unknown. Here, we systematically identified and characterized the expression patterns, evolutionary conservation, genomic features, and internal structures of circRNAs using RNase R-treated libraries from moso bamboo (***Phyllostachys edulis***) seedlings. Moreover, we investigated the biogenesis of circRNAs dependent on both *cis*- and *trans*-regulation. We explored the function of circRNAs, including their roles in regulating microRNA (miRNA)-related genes and modulating the **alternative splicing** of their linear counterparts. Importantly, we developed a customized **degradome** sequencing approach to detect miRNA-mediated cleavage of circRNAs. Finally, we presented a comprehensive view of the participation of circRNAs in the regulation of hormone metabolism upon treatment of bamboo seedlings with GA and NAA. Collectively, our study provides insights into the biogenesis, function, and miRNA-mediated degradation of circRNAs in moso bamboo.

## Introduction

Since the circular genomes of plant viroids [1] and hepatitis delta virus [2] were discovered, it has become clear that circular RNAs (circRNAs) comprise a large class of covalently closed circular molecules that lack 5′ and 3′ ends. Circular intronic RNAs (ciRNAs) and exonic circRNAs (ecircRNAs) are generated via intron lariat debranching and back-splicing of exons, respectively [3], [4]. Although the existence of circular transcripts has been known for at least 40 years [1], their importance has been likely underestimated due to their low abundance [5]. Recently, more comprehensive investigation of such molecules has resulted from the ability to generate libraries enriched for circRNAs and from the availability of circRNA-specific bioinformatics algorithms [6], [7].

Back-splicing is much less completely understood compared to canonical splicing. Although most circRNAs in mammals [3] and *Caenorhabditis elegans* [8] are processed from internal exons with long flanking introns containing inverted complementary sequences (ICSs) [3], [7], [9], formation of circRNAs in other species such as *Drosophila melanogaster* [10] and *Oryza sativa* [11] does not require RNA pairing across flanking introns. In addition to such *cis*-regulation, recent studies have revealed that inhibiting RNA-binding proteins (RBPs) could directly alter circRNA expression [12]. For instance, RBPs such as interleukin enhancer binding factor 3 (ILF3), nuclear factor 90 (NF90) isoform, DExH-box helicase 9 (DHX9), double-stranded RNA-specific adenosine deaminase (ADAR1), and KH domain containing RNA binding (QKI), regulate the formation of ecircRNAs [8], [13], [14], [15], [16]. Splicing factors such as FUS RNA binding protein (FUS), heterogeneous nuclear ribonucleoprotein L (HNRNPL), and QKI, regulate circRNA expression by binding to the flanking introns of circRNAs [13], [17]. In addition, depleting core spliceosomal components such as SF3A and SF3B caused a marked increase in circRNA levels [12]. Thus, RBPs have an important role in regulating the biogenesis of circRNAs.

In addition to their biogenesis, the decay of circRNAs directly influences their accumulation levels. Endonuclease RNase L initiates cleavage of circRNAs. Some circRNAs containing microRNA (miRNA) binding sites such as CDR1 antisense RNA (*CDR1as*) may be degraded by AGO2-mediated cleavage [18]. However, the mechanism of the degradation for most circRNAs remains elusive. Regarding circRNA function, both transcription and splicing of host genes can be modulated by specific circRNAs [4], [19]. For example, *circSEP3* has been proposed to form an RNA:DNA hybrid (R-loop) with its cognate DNA locus in the nucleus; and this R-loop alters exon skipping of linear *SEP3*, which influences floral homeotic phenotypes in *Arabidopsis thaliana* [20].

Thus far, the biogenesis, functions, and decay of circRNAs have not been well understood. In this study, we systematically identified and characterized circRNAs via RNase R-treated RNA sequencing (RNA-seq) libraries from moso bamboo seedlings, with or without gibberellin (GA) and auxin (1-naphthaleneacetic acid, NAA) treatments. Furthermore, we investigated whether circRNAs might regulate the splicing of their corresponding linear RNAs due to R-loop structures. Importantly, we took advantage of a modified protocol for degradome sequencing to detect several miRNA-mediated cleavage events. Finally, we determined that expression of circRNAs could not only be modulated dynamically by GA or NAA hormones but also might affect several hormone-related genes in moso bamboo.

## Results

### Profile of circRNAs in moso bamboo seedlings

**Figure 1:**
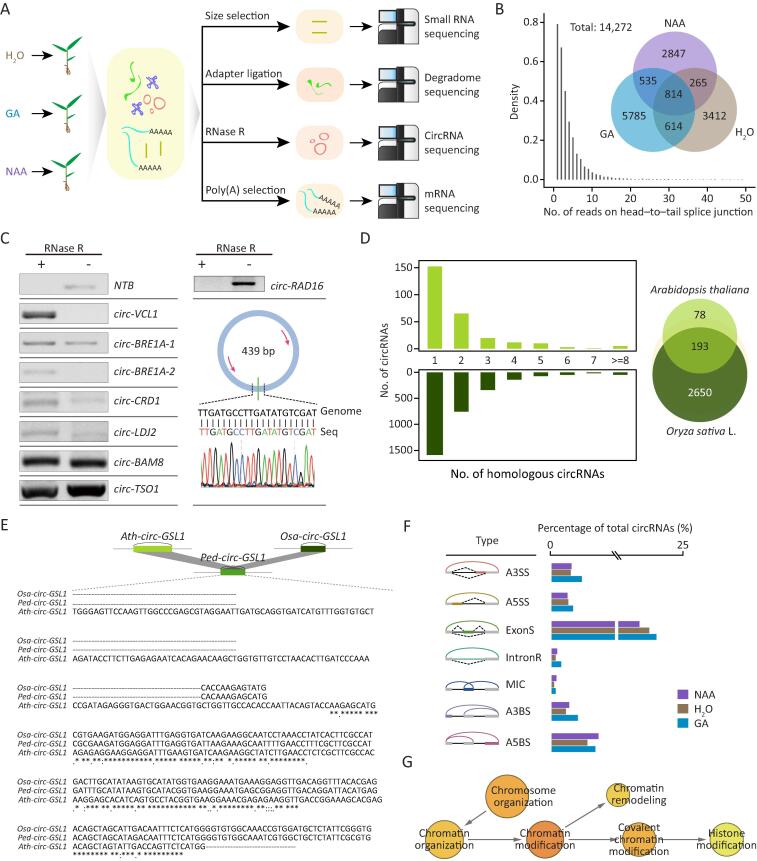
**Characterization of circRNAs in moso bamboo** **A.** Flow chart for multi-omics sequencing including mRNA sequencing, circRNA sequencing, degradome sequencing, and small RNA sequencing upon hormone treatment. **B.** Venn diagram showing the number of circRNAs detected in different samples (upper panel). The density plot of back-splicing reads supporting circRNAs (lower panel). **C.** Validation of circRNAs using RT-PCR after RNase R treatment with linear RNA (*NTB*) as control (left panel). Sanger sequencing validated back-splicing junctions of *circ-RAD16*, which was not resistant to RNase R (right panel). **D.** Number (left) and overlap (right) of circRNAs in *Arabidopsis thaliana* and *Oryza sativa* L. that show homology to circRNAs in moso bamboo. **E.** Multiple sequence alignment of conserved *circ-GSL1*. Asterisk symbols indicate highly conservative nucleotides. **F.** Diagrams of different AS types (left panel) and percentage of these circRNAs (right panel). Gray bars and black lines represent exons and introns, respectively. Dotted curves and colored bars indicate AS events. Colored arced lines represent back-splicing (circularization). **G.** GO enrichment analysis of circRNAs with AS events. Darker orange node color indicates more significant *P* values. The circle size is proportional to the number of genes enriched in the terms. The arrows represent hierarchical relations between GO terms. circRNA, circular RNA; AS, alternative splicing; GO, Gene Ontology; A3SS, alternative 3′ splice site; A5SS, alternative 5′ splice site; ExonS, exon skipping; IntronR, intron retention; A3BS, alternative 3′ back-splice site; A5BS, alternative 5′ back-splice site; MIC, mutually inclusive circRNA; mRNA, messenger RNA; RT-PCR, reverse transcription-polymerase chain reaction; GA, gibberellin; NAA, 1-naphthaleneacetic acid.

To enrich circRNAs in moso bamboo seedling samples, total RNAs from seedlings treated with double-distilled water (ddH_2_O), NAA, or GA were incubated with RNase R and Ribo-Zero. Sequences were then generated from three biological repeats (Figure 1A). In total, we detected 5105, 4461, and 7748 putative circRNAs from ddH_2_O, NAA, and GA treatments, respectively (Figure 1B; Table S1). To independently test whether these sequences represent circRNAs, we carried out reverse transcription-polymerase chain reaction (RT-PCR)-based sequence validation with divergent primers for eight randomly selected circRNAs after RNase R treatment. This validation demonstrated that most circRNAs were indeed resistant to exonuclease degradation. The exception was *circ-RAD16*, which was further discovered to include back-spliced junction sites by Sanger sequencing (Figure 1C). Thus, we concluded that *circ-RAD16* is an authentic circRNA.

We identified conserved circRNAs by comparing our data with published data of other species [21]. As shown in Figure 1D, many circRNAs of moso bamboo were homologous to circRNAs of *A*. *thaliana* or *O*. *sativa*. As expected, circRNAs in bamboo exhibited more homology to those of *O*. *sativa* (2843, 19.9% of all circRNAs) than to *A*. *thaliana* (271, 1.9% of all circRNAs). Notably, 193 (1.3% of all circRNAs) were simultaneously detected in all three species, which included *circ-GSL1* (Figure 1E**)** generated from the gene encoding callose synthases 1 [22]. Enriched Gene Ontology (GO) terms for these evolutionarily conserved circRNAs included rhythmic processes, transporter activity and protein-containing complexes (Figure S1).

The complexity and diversity of circRNAs are further increased by alternative back-splicing [6], [7], [23], which includes the alternative 3′ splice site (A3SS), alternative 5′ splice site (A5SS), exon skipping (ExonS), intron retention (IntronR), alternative 3′ back-splice site (A3BS), and alternative 5′ back-splice site (A5BS), as well as mutually inclusive circRNA (MIC). circRNAs generated by these seven types of alternative back-splicing were identified from all samples by CIRCexplorer2 [7] (Figure 1F). Among the four alternative splicing (AS) types (A3SS, A5SS, ExonS, and IntronR) that generated the different ecircRNA transcripts with same back-splicing sites, IntronR is the most prevalent in linear transcripts of plants, whereas ExonS was the most prevalent from the interior of ecircRNAs. The other three types (A3BS, A5BS, and MIC) generated different ecircRNAs with different back-splicing sites; and A5BA accounted for the largest number of AS events among these three types. Enriched GO terms for these types of ecircRNAs were involved in diverse biological functions, such as chromosome organization, chromatin remodeling, and histone modification (Figure 1G).

### The biogenesis of circRNAs is influenced by *cis*- and *trans*-regulation

**Figure 2:**
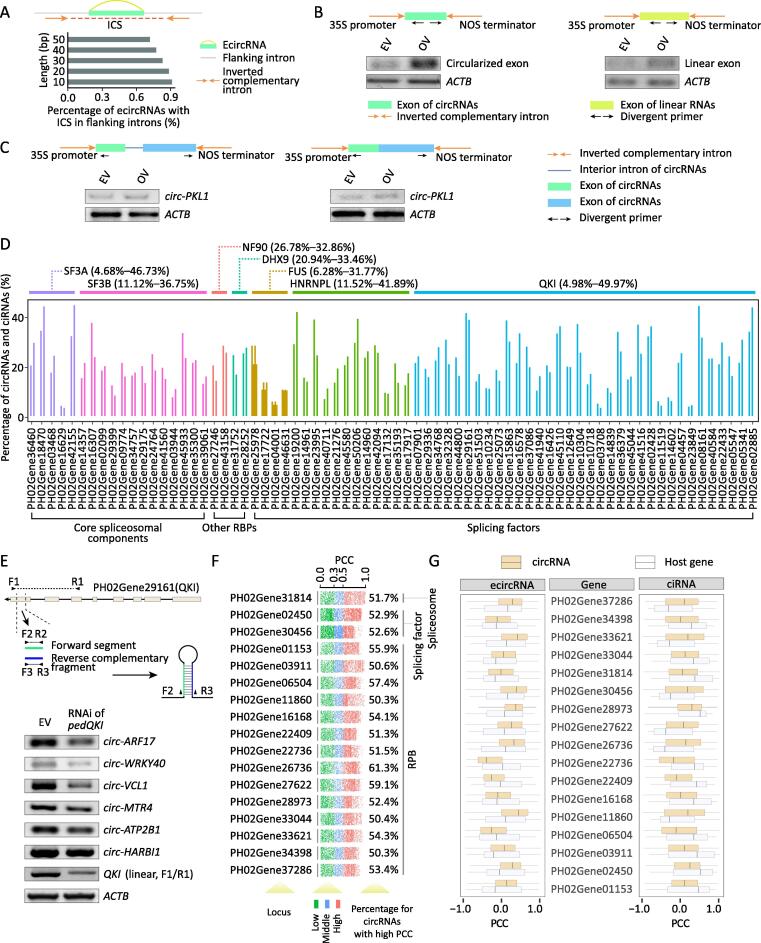
**Biogenesis of circRNAs in moso bamboo** **A.** Distribution of ICSs (brown arrows) in flanking introns of ecircRNAs. Circularized exon and flanking intron are indicated by green bar and gray line, respectively. **B.** Schematic drawings show expression vectors including flanking ICSs (brown arrows), circularized exon (green bar), and linear exon (yellow bar). PCR primers for circRNAs are indicated by black arrows. Semi-quantitative RT-PCR in the lower panel shows circularization efficiency for circularized exon and linear exon, respectively. **C.** Experimental detection of the function of introns in multiple exon circularization of *circ-PKL1*. Schematic drawings of expression vectors including or excluding interior introns. Semi-quantitative RT-PCR in the lower panel shows circularization efficiency of *circ-PKL1* with or without interior intron. **D.** Percentage of circRNAs showing high PCC (*r* ≥ 0.5 or *r* ≤  −0.5) with proteins SF3A, SF3B, FUS, HNRNPL, QKI, NF90, and DHX9. **E.** RNAi knockdown of *PedQKI*. Schematic drawing in the upper panel shows RNAi expression vector to knock down expression of *PedQKI*. The lower panel shows PCR-based validation of the expression of six randomly selected circRNAs in the *QKI* RNAi sample. *ATCB* is a housekeeping gene used as a control.  **F.** PCCs between circRNAs and 17 proteins including 1, 2, and 14 core spliceosomal factors, splicing factors, and other RBPs, respectively. Number in the right panel indicates percentage of circRNAs with high PCC to those proteins. **G.** Distribution of PCCs between ecircRNAs/ciRNAs and linear RNAs generated from the same host genes. ecircRNA, exonic circRNA; ICS, inverted complementary sequence; EV, empty vector; OV, overexpression vector; NOS, nopaline synthase; PCC, Pearson correlation coefficient; RNAi, RNA interference; RBP, RNA-binding protein; ciRNA, circular intronic RNA.

Although ecircRNA-producing loci in moso bamboo usually had longer flanking introns than control introns (Figure S2), the ecircRNAs themselves lacked obvious flanking intronic pairing sequences (Figure 2A), consistent with previous studies [3]. However, ecircRNA production can be driven by long artificial flanking inverted complementary introns [11]. To test the correlation of ICSs with the biogenesis of ecircRNAs, we cloned a 139-bp inverted complementary flanking intron with a representative exon from *CSLA1*, as a representative ecircRNA, and from the third linear exon of *NRT1*, which does not give rise to ecircRNAs (Figure 2B). Semi-quantitative RT-PCR revealed that both the circularized exon and the linear exon in the overexpression vectors exhibited much higher circularization efficiency than that in the wild type, suggesting that RNA pairing with ICSs could enhance back-splicing efficiency of ecircRNAs. In particular, the linear exon from *NRT1* also could be induced into ecircRNAs by ICSs as flanking introns. To determine whether interior introns from multiple circularized exons were involved in the biogenesis of ecircRNA, we constructed *circ-PKL1* by including or excluding the interior intron. The presence of the interior intron did not lead to much difference in the rate of ecircRNA production (Figure 2C), suggesting that interior introns might not be key regulatory elements for the biogenesis of *circ-PKL1*.

Core spliceosomal components, splicing factors, and some RBPs have been also reported to participate in circRNA regulation [7], [14], [24], [25]. To further explore the interplay between circRNAs and these proteins, we performed sequence similarity analysis and identified 124 putative core spliceosomal components, 92 splicing factors, and 1132 other RBPs excluding splicing factors and core spliceosomal components in moso bamboo. We also performed RNA-seq to calculate accumulation levels for linear transcripts (Figure 1A). Pearson correlation coefficients (PCCs) were calculated based on levels of circRNAs [reads per million (RPM)] from circRNA sequencing and expression of RBP genes from RNA-seq [fragments per kilobase of exon model per million mapped fragments (FPKM)]. The levels of circRNAs were correlated (either positively or negatively; *r* ≥ 0.5 or *r* ≤ −0.5) with the transcript levels for 44, 57, and 497 of core spliceosomal components, splicing factors, and other RBPs, respectively (Figure S3A and B; Table S2).

We focused on analyzing seven RBPs (SF3A, SF3B, NF90, DHX9, FUS, HNRNPL, and QKI) that have been found to modulate the production of circRNAs in other species [12], [13], [14], [15], [16], [17]. SF3A and SF3B, as core spliceosomal components, were encoded by 5 and 13 homologous genes in bamboo, respectively, and showed correlation in levels with 4.68%–46.73% and 11.12%–36.75% of circRNAs (Figure 2D). In mammals, NF90 and DHX9 contain double-stranded RNA (dsRNA) binding domains (dsRBDs) and facilitate circRNA formation by directly binding inverted repeated *Alus* (IR*Alus*) [15], [16]. Although the *Alu* element is rare in moso bamboo, NF90 and DHX9 exhibited correlation with 26.78%–32.86% and 20.94%–33.46% of circRNAs, respectively (Figure 2D). In addition, the levels of 6.28%–31.77%, 11.52%–41.89%, and 4.98%–49.97% of circRNAs (Figure 2D) were strongly correlated with those of transcripts encoding 4, 12, and 34 proteins homologous to splicing factors FUS, HNRNPL, and QKI, respectively. A striking example was *PedQKI* (PH02Gene29161) (Figure S4), which encodes a conserved KH domain and shows negative or positive correlation with expression of 579 ecircRNAs and 423 ciRNAs (a total of 49.97% of selected circRNAs), respectively. Although expression of *circ-HARBI1* and *circ-ATP2B1* was not markedly altered, knockdown of *PedQKI* decreased the abundance of *circ-ARF17*, *circ-WRKY40*, *circ-VCL1*, and *circ-MTR4* (Figure 2E).

Notably, 17 previously unreported proteins, namely, 1 core spliceosomal factor, 2 splicing factors, and 14 other RBPs, were more highly correlated with circRNAs than the above seven RBPs (Figure 2F). We further explored the correlation between the expression of these 17 RBPs and of the host genes that generated these circRNAs. We found that the circRNAs and their host genes exhibited distinct correlation patterns (Figure 2G). Taken together, these results suggest that core spliceosomal factors, splicing factors, and other RBPs might serve as regulators of the biogenesis of abundant circRNAs. However, this analysis only identified potential RBP regulators by measure of linear dependence/correlation relationships using PCCs between the expression of circRNAs and genes for RBPs. Thus, experimental analysis of RBP–RNA interaction will be required in the future to identify direct regulators of specific circRNA biogenesis by these RBPs.

### circRNA is involved in the regulation of AS by R-loop structures

**Figure 3:**
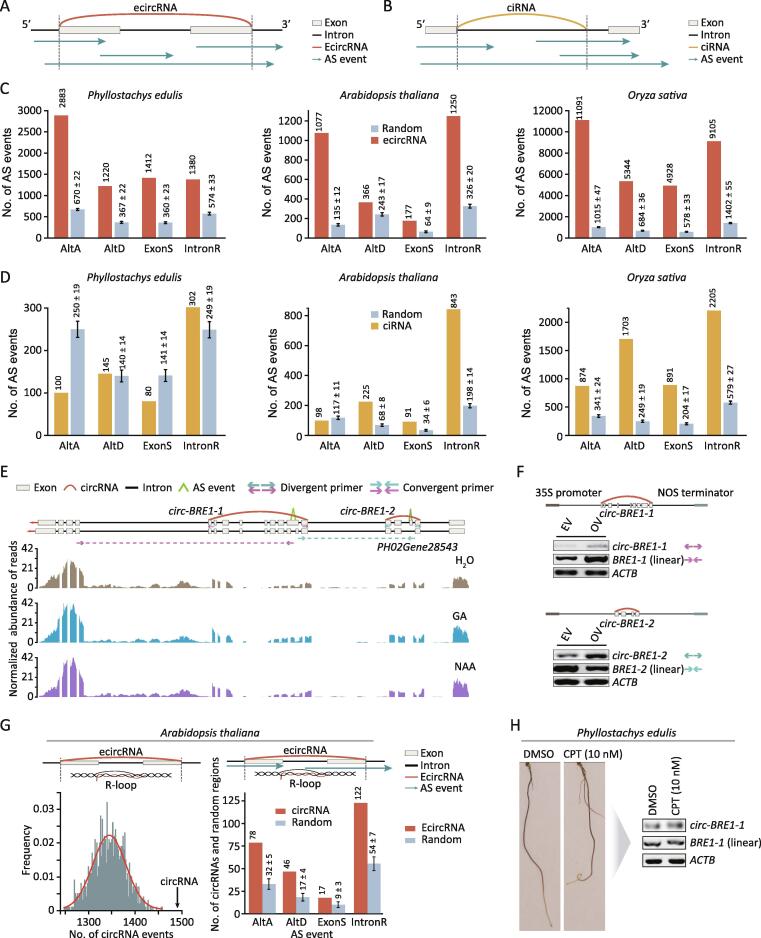
**circRNAs regulate AS in moso bamboo** **A.** Overview of overlapping regions between AS events and ecircRNAs. White bars indicate exons, black bars indicate introns, blue arrows indicate AS events, and colored arced lines indicate back-splicing junctions. **B.** Overview of overlapping regions between AS events and ciRNAs. **C.** The histograms show the number of AS events in the transcribed regions of ecircRNAs (brown bars) and random RNA segments (gray bars) in three species. **D.** The histograms show the number of AS events in the transcribed regions of ciRNAs (light yellow bars) and random RNA segments (gray bars) in three species. **E.** Visualization of IntronR and ExonS events in transcribed regions of *circ-BRE1* and *circ-BRE2*, respectively. **F.** RT-PCR validation of *circ-BRE1-1* and *circ-BRE1-2* and their corresponding AS events, linear *BRE1-1* and linear *BRE1-2*. Divergent arrows represent divergent primers, and convergent arrows represent convergent primers. Linear RNA of *ACTB* was used as a control. **G.** The histogram plot shows the overlap between R-loop and AS events in transcribed regions of ecircRNAs. Black arrow in the left panel indicates the observed number of ecircRNAs located in R-loop regions. **H.** Root phenotype upon DMSO and CPT treatment (left panel). Semi-quantitative RT-PCR shows the expression of *circ-BRE1-1* and linear *BRE1-1* upon CPT treatment (right panel). R-loop, RNA:DNA hybrid; AltA, alternative acceptor; AltD, alternative donor; DMSO, dimethyl sulfoxide; CPT, camptothecin.

Our previous study revealed that overexpressing *circ-IRX7* in *Populus trichocarpa* decreased the rate of IntronR in the linear *IRX7* counterpart [26]. In this study, we used our RNA-seq and published data [27], [28] to identify AS events in bamboo, *A*. *thaliana*, and *O*. *sativa*. We subsequently detected the locations of AS events within ecircRNAs and ciRNAs across the three species (Figure 3A and B; Table S3). To further validate if AS preferentially occurred within ecircRNAs, we randomly extracted the same number of messenger RNA (mRNA) segments without producing ecircRNAs and identified locations of AS events within these segments. The AS within the simulated random RNA segments had considerably lower frequencies than that observed within ecircRNAs (Figure 3C). This trend was also observed in *A*. *thaliana* and *O*. *sativa* (Figure 3C), which demonstrated that the elevated frequency of AS events within ecircRNAs might be conserved in different species. However, AS events located in the ciRNAs did not exhibit this trend (Figure 3D).

PCCs between circRNAs and four types of AS events were calculated using their expression profiles according to circRNA sequencing (RPM) and RNA-seq (normalized reads that span splicing junctions), respectively. Approximately 19%–48% of circular transcripts in three species showed positive relationships with AS events (Table S3). To further validate the regulation of splicing by circRNAs, we selected *circ-BRE1-1* and *circ-BRE1-2*, which overlapped with ExonS and IntronR events from *E3 ubiquitin-protein ligase BRE1-like 1*, respectively (Figure 3E). Indeed, overexpression of *circ-BRE1-1* and *circ-BRE1-2* considerably changed the number of events in which the long isoforms of IntronR (linear *BRE1-1*) and ExonS (linear *BRE1-2*) were formed (Figure 3F), which indicates that circRNAs can modulate the AS events of their linear counterpart.

It has been shown that the presence of *circSEP3* was derived from an exon of *SEP3*, which could result in exon skipping of its linear transcripts via an R-loop structure [20]. We therefore identified R-loop structures including AS events in transcribed regions of ecircRNAs by integrating circRNA, RNA-seq [28], and R-loop data [29] from *A*. *thaliana*. Notably, transcribed regions of ecircRNAs had a considerably greater frequency of R-loop events compared with random regions (Figure 3G). Moreover, R-loop events were considerably more enriched in AS events than in random regions (Figure 3G). Camptothecin (CPT) is a TOP1 inhibitor that promotes R-loop accumulation in *A*. *thaliana* [30]. We treated seedlings with dimethyl sulfoxide (DMSO) or CPT (10 nM), which revealed that seedlings treated with CPT had enhanced the abundance of *circ-BRF1-1* and long isoforms of IntronR (linear *BRE1-1*) in the transcribed regions of *circ-BRF1-1* (Figure 3H). Collectively, these findings indicated that circRNAs could regulate the AS frequency of their precursor transcripts, which may be related to R-loop structure formation by circRNA:DNA hybrids.

### circRNA is involved in the regulation of miRNA/siRNA-related genes

**Figure 4:**
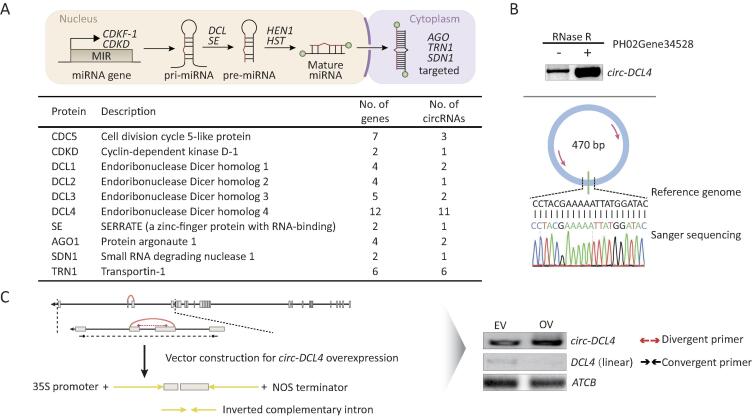
**circRNAs regulate miRNA-associated genes in moso bamboo** **A.** The upper panel shows the miRNA-associated genes involved in several major steps in miRNA biogenesis and modes of action in plants. The table in the lower panel shows the number of miRNA-associated genes and their corresponding circRNAs. **B.** RT-PCR and Sanger sequencing for validation of *circ-DCL4* with RNase R treatment. **C.** The left panel shows the vector construction for *circ-DCL4* and linear *DCL4*. RT-PCR validation in the right panel shows the overexpression of *circ-DCL4* and expression of linear *DCL4*. miRNA, microRNA; HEN1, small RNA 2′-*O*-methyltransferase; CDKF, cyclin-dependent kinase F-1; HST, shikimate *O*-hydroxycinnamoyltransferase.

*circAGO2* is generated from *AGO2* and represses AGO2/miRNA-mediated gene silencing [31]. In total, 163 miRNA-related genes were identified by sequence similarity analysis, and 79 miRNA-related genes, including *AGO1* and *SDN1*, were identified as host genes of circRNAs in moso bamboo (Figure 4A; Table S4). We selected *circ-DCL4*, which originates from *DCL4*, for further validation. An RNase R exonuclease experiment revealed the resistance to exonuclease, and sequencing also validated the back-splice sites in *circ-DCL4* (Figure 4B). Notably, overexpressing of *circ-DCL4* tended to decrease the expression level of its linear RNA (linear *DCL4*) (Figure 4C), which might explain its effect on the biogenesis of miRNAs and small interfering RNAs (siRNAs).

### circRNA is translatable in moso bamboo

In this study, we developed a computational pipeline for detecting translatable open reading frames (ORFs) of circRNAs (cORFs) using proteomics (Figure S5A). Moreover, we identified upstream ORFs (uORFs) and downstream ORFs (dORFs) of linear transcripts. Annotated coding sequence (CDS) region for each gene was regarded as primary ORF (pORF). As shown in Figure S5B, a small proportion of cORFs, uORFs, and dORFs showed sequence similarity to entries in Non-Redundant Protein Sequence Database (NR), Database Of Plant Small ORFs (PsORF), and Universal Protein Resource (UniProt). For instance, a conserved cORF derived from *circ-GLO5* was homologous to small ORFs in plants from the PsORF database [32], which indicated that these circRNAs might encode functional peptides (Figure S5C). As the third step, all ORFs were used as a search library for liquid chromatography–mass spectrometry/mass spectrometry (LC–MS/MS)-based proteomics to identify unique peptide evidence for each ORF. In total, 538 cORFs from 536 circRNAs (approximately 7.1% of all 7554 circRNAs) were predicted to be translatable based on proteomics evidence (Figure S5D; Table S5). For example, *circ-P4H-1* generated a detectable protein with a length of 289 aa spanning the back-splicing site with the evidence of a unique peptide (Figure S5E).

### miRNA-mediated cleavage of circRNAs

**Figure 5:**
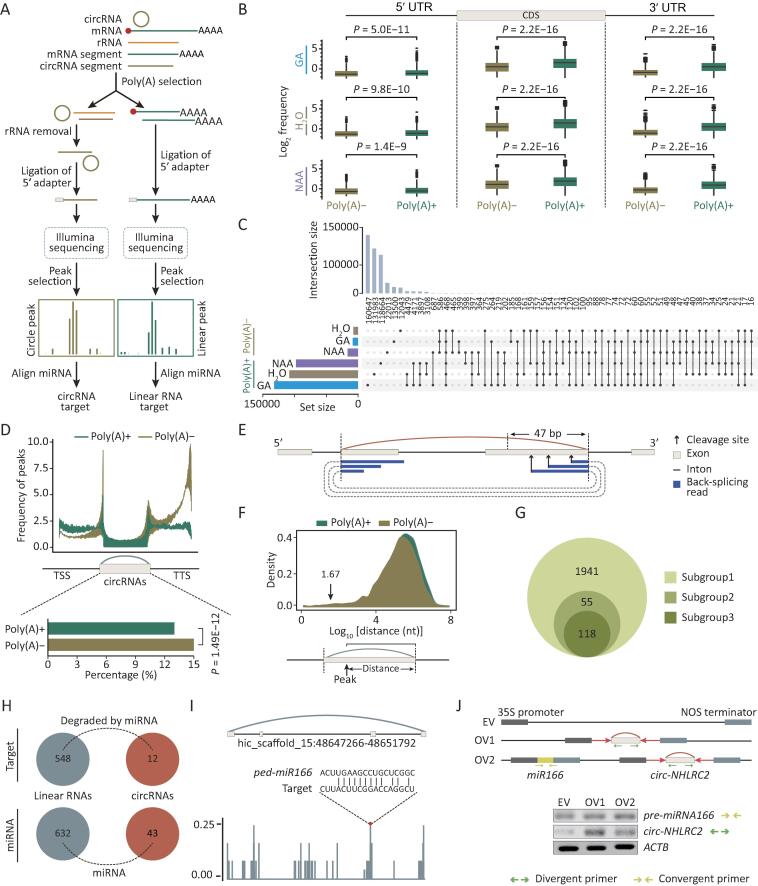
**miRNA-mediated cleavage of circRNAs in moso bamboo** **A.** Flow chart for construction of customized degradome libraries for enriching the decaying circRNA without poly(A) tails. **B.** The box line plot shows the distribution of reads in 5′ UTR, CDS, and 3′ UTR of the gene from poly(A)+ and poly(A)− degradome libraries. **C.** The UpSet plot shows the intersection of cleavage sites among different types of libraries and different hormone treatments. The histogram plot in the upper panel shows the number of cleavage sites shared by different libraries. The histogram plot in the lower panel and the line plot in the right panel show total cleavage sites from each library and the combination of different libraries. **D.** Plot in the upper panel shows the distribution of cleavage sites from two types of libraries in genes divided into three groups: the upstream region of circRNA body, downstream region of circRNA body, and circRNA body. Bar in lower panel shows the percentage of cleavage sites from two types of libraries in transcript regions of circRNAs. **E.** Schematic overview of decaying reads of circRNAs spanning back-splicing sites. Gray bars represent exons, black lines represent introns, black arrows represent cleavage sites, and colored arced lines represent back-splicing. Blue bars and dash arced lines indicate the mapped back-splicing junction reads. **F.** Length distribution between cleavage sites and the 3′ end of transcribed regions of circRNAs. **G.** Venn diagram shows the number of three subgroups. Subgroups 1, 2, and 3 represent the number of circRNAs including cleavage sites based on degradome sequencing, the number of circRNAs with a distance of < 47 nt between cleavage sites and the 3′ end of transcriptional regions of circRNAs, and the number of circRNAs determined by degradome reads spanning back-splicing sites. **H.** Number of circRNAs and linear RNAs including miRNA-mediated cleavage. **I.** Schematic overview of *miR166*-mediated cleavage sites in *circ-NHLRC2*. **J.** The upper panel shows the vector construction for overexpressing *circ-NHLRC2* only (OV1) or both *circ-NHLRC2* and *miR166* (OV2). The lower panel indicates the expression of *circ-NHLRC2* and *miR166* detected by RT-PCR. Divergent and convergent arrows represent divergent and convergent primers, respectively. rRNA, ribosomal RNA; CDS, coding sequence; UTR, untranslated region; TSS, transcription start site; TTS, transcript termination site.

Endonucleases RNase L has been reported to decay circRNAs globally in animals [33]. Sequence similarity analysis of RNase L from 67 animals revealed a high identity (on average 80.5%) and alignment length (on average 727 aa). By contrast, we did not identify RNase L sequences in 100 plants including moso bamboo (Figure S6A and B), suggesting that the decay of circRNAs in plants is not mediated by RNase L. Given that miR-671 was identified as a key regulator in the decay of *CDR1as* [18], we hypothesized that miRNAs may also be potential factors contributing to the decay of circRNAs in plants. We began by sequencing nine small RNA libraries constructed from the same material as circRNAs (Figure 1A). We detected 823 mature miRNAs including 164 conserved miRNAs and 659 variant miRNAs, which clustered to form 43 miRNA families (Figure S7A; Table S4). Conventional degradome sequencing [34] could not detect the cleavage of circular molecules due to the absence of 3′ poly(A) tails of circRNAs. Therefore, we modified the protocol for generating degradome libraries so that we could identify miRNA-mediated cleavage of circRNAs (Figure 5A). After poly(A) selection, total RNAs were separated into poly(A)− and poly(A)+ transcripts. Subsequently, the poly(A)− transcripts were subjected to ribosomal RNA (rRNA) depletion followed by immediate ligation to 5′ RNA adapters to enrich the cleavage transcripts of circRNAs including free 5′ monophosphate, whereas the 5′ monophosphate of poly(A)+ transcripts was directly ligated to the 5′ RNA adapter. Finally, these two types of degradome libraries were prepared by reverse transcription and sequenced. As expected, transcript regions including the 5′ untranslated region (5′ UTR), CDS, and 3′ UTR were more enriched with free 5′ monophosphate reads in the poly (A)+ library than in the poly (A)− library (Figure 5B). Consistent with previous observations that the cleavage transcripts were biased toward the 3′ end of mRNAs [35], the poly(A)+ library also tended to generate more reads including free 5′ monophosphate in the 3′ UTR than in the 5′ UTR. However, the poly(A)− library did not exhibit this tendency (Figure S7B).

We further developed a computational strategy to identify miRNA cleavage events in circRNAs, termed “degradome peaks in poly(A)− transcripts” (Figure S7C). In brief, degradome sequencing reads were first aligned to the genome using Bowtie 2 [36] to detect accumulated cleavage events termed “degradome peaks”. As indicated in Figure 5C, degradome peaks within annotated transcript regions from each poly(A)+ library were considerably higher than those in the poly(A)− library from the same samples. The distribution of degradome peaks was considerably enriched in upstream and downstream regions of circRNAs, regardless of whether the peaks originated from poly(A)+ or poly(A)− libraries (Figure 5D, upper). We further compared the distribution of degradome peaks between poly(A)− and poly(A)+ libraries in host genes that generated circRNAs. We observed that the frequency of degradome peaks from the poly(A)− library (approximately 15%) was slightly higher (*P* = 1.49E−12, Fisher’s test) than that from the poly(A)+ library (approximately 13%) in the region of circRNAs (Figure 5D, lower).

We calculated the distance from peaks to back-splicing sites to search for the decaying peaks spanning back-splicing sites (Figure 5E and F). The degradome reads from these peaks were then aligned to the 50 upstream and downstream nucleotides of back-splicing sites (Figure S7D). In total, degradome peaks from 118 circRNAs were revealed by degradome reads spanning back-splicing sites (Figure 5G). These results collectively suggest that our customized libraries and computational pipeline could effectively identify the decaying sites of poly(A)− transcripts, particularly for circRNAs.

We further identified degradome peaks in the poly(A)− library originating from the miRNA-mediated cleavage of circRNAs. The 25 upstream and downstream nucleotides of specific degradome peaks were aligned to mature miRNAs by RNAplex [37] to identify candidate miRNA cleavage sites in the ±1-bp region of degradome peaks. Overall, 12 circRNAs and 548 linear RNAs were identified as the cleavage transcripts mediated by 43 and 632 miRNAs, respectively (Figure 5H; Table S6). For instance, we observed that *ped-miRNA166* mediated cleavage in *circ-NHLRC2* (Figure 5I). For further validation, we overexpressed *circ-NHLRC2* (OV1) and both *circ-NHLRC2* and *miR166* (OV2) (Figure 5J**)**. Semi-quantitative RT-PCR revealed that the expression of *circ-NHLRC2* was considerably increased in protoplasts transformed and overexpressing only *circ-NHLRC2* (OV1). By contrast, *circ-NHLRC2* exhibited a decreased tendency in the OV2 vector carrying both *circ-NHLRC2* and *miR166* (Figure 5J). Taken together, these observations suggest that miRNAs could contribute to the degradation of circRNAs in moso bamboo.

### circRNA expression is modulated by GA and NAA

**Figure 6:**
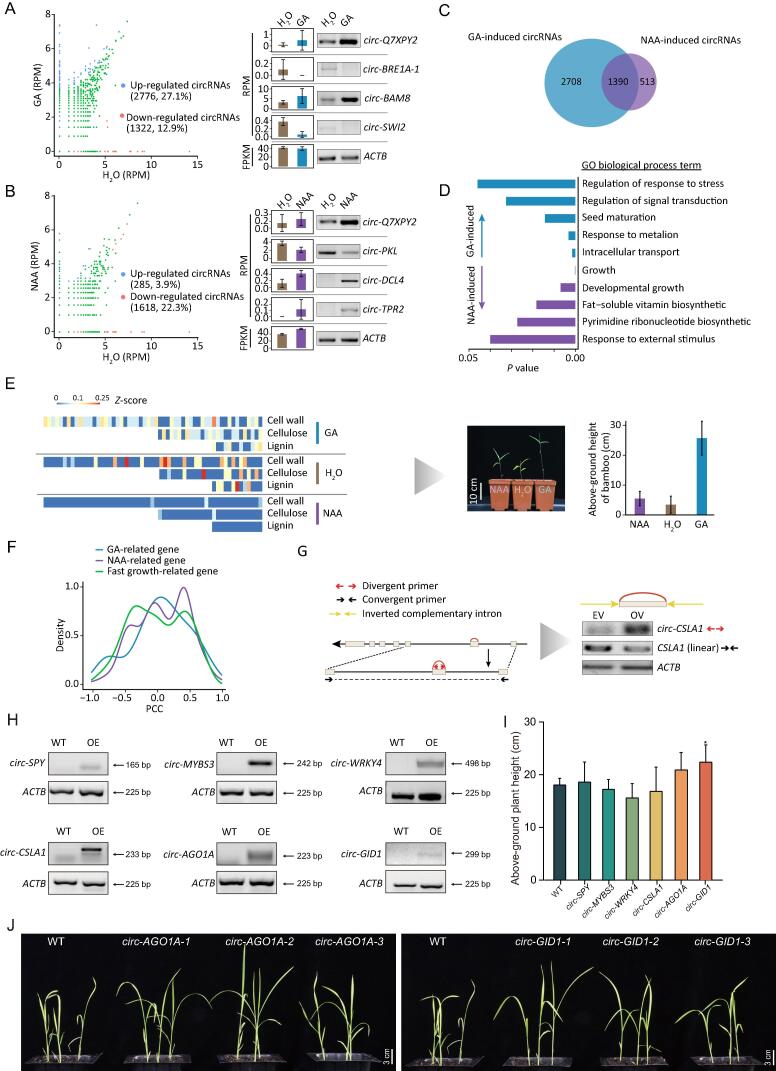
**Hormone-induced circRNAs in moso bamboo** **A.** The scatterplot in the left panel shows RPMs for H_2_O (X-axis) and GA treatments (Y-axis). Semi-quantitative PCR using divergent primers in the right panel validated the differential expression data of circRNAs in response to GA treatments with linear *ACTB* as internal reference gene using convergent primers. **B.** The scatterplot shows the differential levels of circRNAs in response to NAA, and semi-quantitative PCR shows the validation of the levels of circRNAs based on sequencing. **C.** Venn diagram shows overlapping and unique differential circRNAs in response to GA and NAA. **D.** The histogram plot shows the top 10 GO terms enriched for hormone-induced circRNAs. **E.** Heatmap showing expression levels of circular transcripts related to cell wall, cellulose, and lignin. Red or blue represents high and low abundance of circRNAs, respectively. Image and bar chart in the right panel show the phenotype and height of seedlings upon GA and NAA treatments after 2 weeks. **F.** The PCCs of circRNAs and their host linear RNAs related to fast growth, NAA, and GA. **G.** The vector construction (left panel) and RT-PCR validation (right panel) of *circ-CSLA1* and linear *CSLA1*. Divergent and convergent arrows represent divergent and convergent primers, respectively. **H.** RT-PCR using divergent primers revealed the expression levels of circRNAs in six transformed lines (T1 generation). **I.** The histogram shows the plant height of 24-day-old rice seedlings transformed by *circ-SPY*, *circ-MYBS3*, *circ-WRKY4*, *circ-CSLA1*, *circ-AGO1A*, and *circ-GID1*, respectively. **J.** The phenotypes of plant height of rice seedlings transformed by *circ-AGO1A* and *circ-GID1*. RPM, reads per million; FPKM, fragments per kilobase of exon model per million mapped fragments; WT, wild type; OE, Over expression.

The plant hormones GA and NAA are essential for developmental processes in moso bamboo, including root germination and shoot development [38], [39]. However, whether circRNAs respond to hormones has remained unknown. To perform quantitative analyses of circRNAs, we extracted back-splicing junctions relative to normalized circRNA abundance using RPM. Strikingly, the percentage of up-regulated circRNAs upon GA treatment was one-fold more than that of down-regulated circRNAs (27.1% up-regulated and 12.9% down-regulated), whereas substantially fewer were up-regulated than down-regulated upon NAA treatment (3.9% up-regulated and 22.3% down-regulated) (Figure 6A and B; Table S7). Furthermore, the expression patterns of seven tested circRNAs were found to be consistent with circRNA sequencing analysis using semi-quantitative RT-PCR (Figure 6A and B). For instance, *circ-BRE1-1* from *E3 ubiquitin-protein ligase BRE1-like 1* (PH02Gene28543) displayed much lower levels with GA hormone treatment, whereas NAA treatment resulted in higher levels of *circ-DCL4*. These findings indicate that both GA and NAA treatments induced notable changes and that alteration of circRNA levels was more sensitive to GA than to NAA (Figure 6C). Furthermore, transcript levels of 1390 circRNAs were both modulated by GA and NAA, as exemplified by *circ-Q7XPY2* (Figure 6C). GO enrichment analysis for differentially expressed circRNAs revealed that intracellular transport and response to external stimulus were highly enriched in response to GA and NAA treatment, respectively (Figure 6D).

To further investigate the biological effects of hormone-induced circRNAs, we clustered the expression of circRNAs from different samples. As indicated in Figure S8A, the host genes for 67 considerably differentially expressed circRNAs were annotated as being related to second messengers, cell inclusion, plant organs, and plant hormones, namely, the abscisic acid and cytokinin pathways. Moreover, circRNAs resulting from rapid growth-related genes, including those related to the cell wall, cellulose, and lignin, were modulated in response to GA and NAA, which is consistent with the phenotype of seedling growth after hormone treatment (Figure 6E).

The regulation of concentration gradients for gibberellin and auxin hormones is a key process in plants [40], [41]. In total, 15 GA-related and 87 NAA-related genes were identified as parent genes that could produce circRNAs (Table S7). These circRNAs were likely to mediate biogenesis, function, and transport of gibberellins (Figure S8B) and auxins (Figure S8C). For example, *circ-CPS1* and *circ-PhYUC5* were resulted from PH02Gene47426 (*CPS1*) coding ent-copalyl diphosphate synthase 1 and PH02Gene12216 (*PhYUC5*) coding FMO-like, which are involved in gibberellin and auxin biosynthesis, respectively. PCC analysis between circRNAs (RPM) and their linear RNAs (FPKM) indicated that 89 circRNAs correlated in levels with their corresponding linear RNAs (Figure 6F). A striking example was *circ-CSLA1*, which is derived from *glucomannan 4-beta-mannosyltransferase 1* (*CSLA1*) and is involved in generating the backbone used for galactomannan synthesis by galactomannan galactosyltransferase [42]. *circ-CSLA1* was up-regulated in response to GA treatment. Overexpression of *circ-CSLA1* considerably reduced the levels of its linear RNA (Figure 6G). Collectively, our results indicated that alteration of circRNA expression in response to GA and NAA might also modulate the expression of host genes.

As stable transformation is difficult in moso bamboo, and both bamboo and *O*. *sativa* are Poaceae family members [43], we overexpressed six candidate circRNAs (*circ-SPY*, *circ-MYBS3*, *circ-WRKY4*, *circ-CSLA1*, *circ-AGO1A*, and *circ-GID1*) in rice via *Agrobacterium*-mediated transformation. Stably transformed lines showed successful transgene expression of the six circRNAs (Figure 6H). Among these six transformed lines, *circ-AGO1A* and *circ-GID1* increased plant height in three independent lines (Figure 6I and J, Figure S9), demonstrating that circRNAs have biological roles in plants.

## Discussion

Techniques involving profiling with poly(A)− RNA populations or RNase R enrichment have uncovered global expression of circRNAs [24]. In this study, our comprehensive sequencing of an RNase R-treated library identified 14,272 circRNAs (Figure 1A and B), suggesting that this type of library could enhance the identification of circRNAs. These expanded circRNAs raised the possibility of investigating the expression pattern, evolutionary conservation, and internal structure of circRNAs. Notably, the homologous circRNAs were present in low proportions (Figure 1D), which may be explained by the tissue-specific expression. Consistent with previous findings [6], [7], [14], our identified circRNAs harbored alternative back-splicing, AS events, and MICs (Figure 1F). In total, over 60% of genes with circRNA AS events from CIRI-AS [6] overlapped with those from CIRCexplorer2 [7], indicating that most of these events could be identified by the different methods.

Emerging studies have revealed that ecircRNA formation is influenced by *cis*-regulatory elements such as ICSs flanking exons [3] and *trans*-regulatory factors [7], [14], [24], [25]. Our study strongly indicates that artificial RNA pairing with ICSs could enhance back-splicing efficiency in moso bamboo (Figure 2B), although the exons of circular transcripts lack native flanking intronic pairing sequences (Figure 2A). Intriguingly, overexpression of ecircRNAs with included or excluded interior introns suggests that interior introns contained within multiple circularized exons may not be a key regulatory element for the biogenesis of circRNAs (Figure 2C). However, an extended investigation of the splicing of interior introns in other circRNAs is required to obtain a more comprehensive conclusion.

Several known *trans*-factors reported to participate in circRNA regulation [7], [14], [24], [25] also exhibited strong associations with circular molecules in our samples (Figure 2E). For example, QKI was more closely correlated with 579 ecircRNAs than with 423 ciRNAs. Knockdown analysis further confirmed the alteration in abundance for four selected circRNAs (Figure 2E). However, we still do not know whether there is direct regulation of these RPBs on specific motifs or circRNA transcripts due to the lack of RBP–RNA interaction experiment. It will be interesting to investigate these *trans*-regulatory factors for binding motifs around circRNAs using high-throughput sequencing of RNAs isolated by cross-linking immunoprecipitation (CLIP-seq) [44].

Combining RNase R-treated circRNA libraries with a common RNA-seq library provided us the opportunity to comprehensively explore the relationship between circRNAs and the AS of their linear counterparts. We identified several potential regulatory circRNAs, such as *circ-BRE1-1* and *circ-BRE1-2*, which like *circSEP3* [20], affected the AS of their precursor transcripts in *Phyllostachys edulis*, *A*. *thaliana*, and *O*. *sativa* (Figure 3C–F). Subsequently, we observed that transcribed regions of circRNAs overlapped R-loop events and AS events with considerably greater frequency than random regions (Figure 3G). The abundance of long isoforms (linear *BRF1*) was increased after treatment with TOP1 inhibitor, which functions in promoting R-loop accumulation. This result indicated that the R-loop structure could be a potential AS regulator of host genes. Furthermore, *circ-BRF1* also exhibited the identical trend with linear *BRF1*. However, further research is needed to elucidate the relationships between R-loops, AS, and circRNAs, for example, using knockdown analysis of R-loop and a single-strand DNA ligation-based library preparation technique (ssDRIP-seq) [29], which should reveal whether R-loop accumulation can influence circRNA abundance and thus promote AS at the genome-wide level.

Our data showed that partial circRNAs could originate from miRNA-related genes. A striking sample was *circ-DCL4*, which was validated by RNase R exonuclease treatment and Sanger sequencing (Figure 4B). Overexpression of *circ-DCL4* reduced the expression levels of linear *DCL4* considerably (Figure 4C), which suggested that circular transcripts might participate in miRNA biogenesis, activity, and degradation by modulating the level of miRNA-associated genes. The correlation in levels between circRNAs and 823 mature miRNAs provided evidence for this hypothesis (Table S4). In the future, improvement of *Agrobacterium*-mediated transformation efficiency in moso bamboo would allow work to identify how many miRNAs or siRNAs are changed in expression upon overexpressing these specific circRNAs.

We found that plants appear to lack homologs of human RNase L (Figure S6A and B), which led us to test whether miRNAs contribute to the initiation of degradation of circRNAs, similar to linear RNAs in plants. Degradome sequencing is the gold standard for identifying miRNA-mediated cleavage on a transcriptome-wide scale. However, current efforts have not yet detected miRNA-mediated cleavage of circRNAs using conventional degradome sequencing, largely due to the absence of 3′ poly(A) tail on circRNAs. To overcome this limitation, we modified library preparation for degradome sequencing to enrich the decaying transcripts of circRNA without poly(A) tails (Figure 5A) and developed a computational pipeline to identify the cleavage sites in poly(A)− RNA (Figure S7C). The enrichment and accurate identification of cleavage sites from customized libraries were assessed based on the distribution of degradome reads (Figure 5B–D, Figure S7B) and cleavage transcripts spanning the back-splicing sites (Figure 5E–H and 6D). Ultimately, 12 circRNAs were identified as cleavage transcripts mediated by miRNAs (Figure 5H). Overexpressing *miR166* down-regulated the expression of *circ-NHLRC2* (Figure 5I and J), which provides evidence for miRNA-mediated cleavage of circRNA. However, further investigation is needed to elucidate similarities and differences in miRNA-mediated cleavage mechanisms between circRNAs and linear transcripts, the latter of which involves the major effector endonuclease AGO1 [45].

Quantitative analyses allowed us to identify core circRNAs involved in the response to GA and NAA. A total of 1390 circRNAs modulated by both GA and NAA could function as potential mediators of cross-talk between GA and NAA (Figure 6C). Further investigation of the biological functions of these hormone-induced circular transcripts indicated that these circRNAs might be involved in processes related to plant hormones, second messengers, cell inclusion bodies, and plant organs (Figure S8). Notably, genes related to rapid growth, particularly those affecting the cell wall, cellulose, and lignin, also generated considerably differentially expressed circRNAs, which was consistent with the growth-related phenotypes of the hormone-treated seedlings (Figure 6E). In addition, circRNAs derived from 15 GA-related and 87 NAA-related genes were likely to mediate biogenesis, function, and transport processes of gibberellin and auxin molecules (Figure S8B and C). Together, these findings indicate a link between circRNAs and hormones, although further experiments such as the transgenic expression of circRNAs associated with hormone regulation are required to elucidate the underlying mechanisms in bamboo.

In previous studies, genes from moso bamboo were overexpressed in *A*. *thaliana* and *O*. *sativa.* In total, 54 genes from previous studies have been reported to play key roles based on *Agrobacterium*-mediated transformation [46], [47], [48], [49], [50], [51], [52], [53], [54], [55], [56], [57], [58]. Among these overexpressed genes, we found 12 genes to generate circRNAs in this study (Table S8). For example, *CWINV4*, encoding cell wall invertase, increased plant height and dry weight in *A*. *thaliana* [58] and generated two circRNAs. It will be interesting to investigate the biological role of these circRNAs in bamboo. Here, we overexpressed six candidate circRNAs in rice and observed influences on plant height phenotypes from *circ-AGO1A* and *circ-GID1* (Figure 6J), which suggested that some circRNAs have biological roles. The precise roles of these circRNAs could be investigated in the endogenous bamboo system if *Agrobacterium*-mediated transformation efficiency improves in moso bamboo in the future.

**Figure 7:**
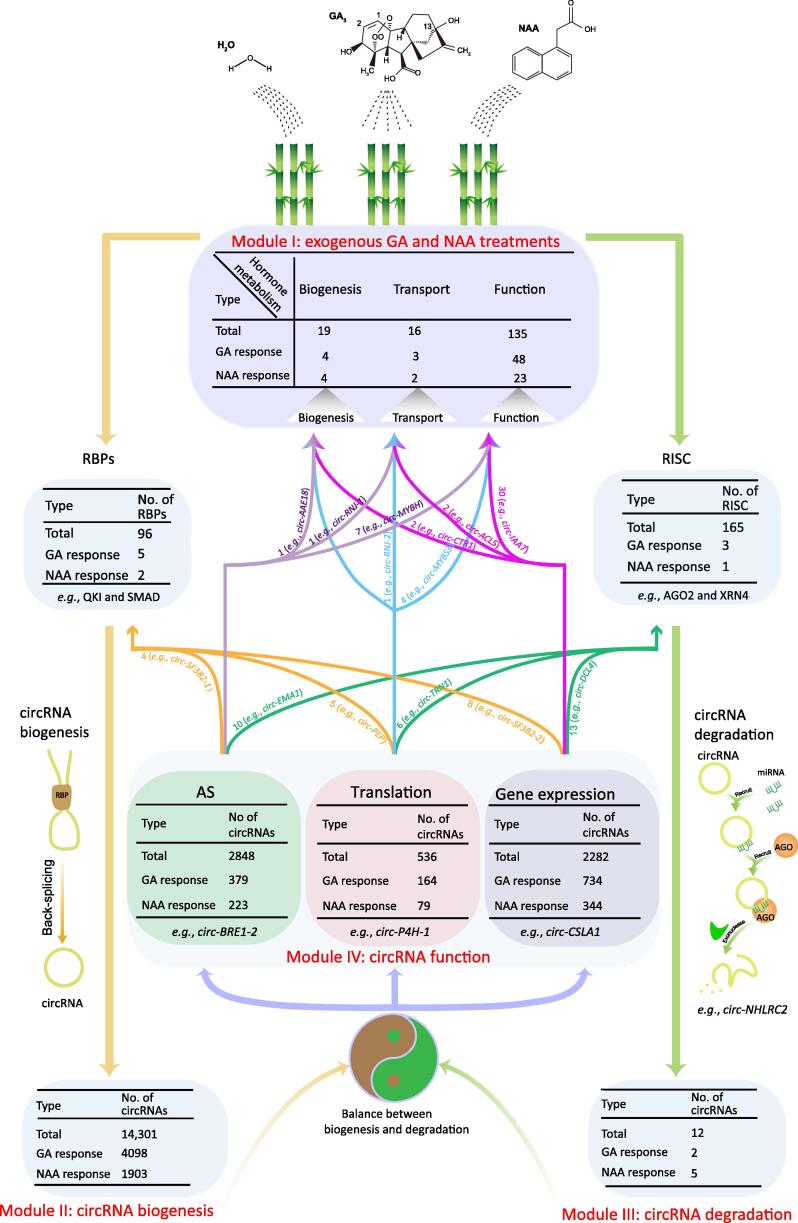
**The potential interplay between hormone and circRNA metabolism** Module I: circRNAs originating from hormone-related genes exhibit dynamic expression upon exposure to GA and NAA. Module II: hormones could affect circRNA biogenesis by regulating several splicing factors. Module III: hormones could regulate the degradation of circRNAs though modulating RSC. Module IV: functional circRNAs might regulate hormone metabolism by regulating splicing and transcription of their linear cognates or generating potential proteins from translatable circRNAs. RISC, RNA-induced silencing complex; RSC, RNA silencing complex.

Overall, our study not only enhances the view of hormone responses in moso bamboo (Figure 7, Module I) but also expands the understanding of circRNAs, including biogenesis (Figure 7, Module II), miRNA-mediated degradation (Figure 7, Module III), and function (Figure 7, Module IV). In particular, our results dissected the interplay between hormones and circRNA metabolism (Figure 7; Table S9). First, the expression levels of circRNAs exhibited high correlation with those of 96 splicing factor genes, including five GA-induced factors and two NAA-induced factors (Figure 7; Table S9), which was consistent with our findings that alteration of circRNA levels upon GA treatment was more sensitive than that upon NAA treatment (Figure 7, Module II: circRNA biogenesis). Second, circRNAs were cleaved by RNA-induced silencing complex (RISC), including three GA-induced RISC and one NAA-induced RISC. We further found that seven circRNAs were subject to miRNA-mediated cleavage in response to hormone treatment, suggesting that hormones might affect the degradation of circRNAs by modulating the expression of RNA silencing complex (RSCs) (Figure 7, Module III: circRNA degradation). The steady-state abundance of circRNAs will depend on the balance between these processes of circRNA biogenesis and degradation. Finally, we revealed potential functions of circRNAs, including regulation of AS, translation, and gene expression (Figure 7, Module IV: circRNA function). In total, 379 GA-induced circRNAs and 223 NAA-induced circRNAs were positively correlated with AS of their host gene, indicating that these differentially expressed circRNAs might alter AS events in response to hormone signals. Notably, several circRNAs, such as *circ-SF3B2-1*, *circ-EMA1*, and *circ-MYBH*, might be involved in the metabolism of both circRNAs and hormones by affecting the splicing of their linear mRNA counterparts (Figure 7).

We further provide evidence that translatable circRNAs might generate functional peptides. We found that translatable circRNAs exhibited dynamic expression upon exposure to hormones. For example, *circ-MYBS* generated a detectable unique peptide, which might play potential roles in regulating hormone metabolism. Moreover, circRNAs could regulate transcription of their parental genes (Figure 7, Module IV: circRNA function). A striking example was *circ-DCL4*, originated from miRNA-related genes, which tended to reduce the accumulation level of its linear RNA. The expression of genes related to hormone metabolism might be regulated by several circRNAs, including *circ-CTR1*, *circ-ACL5*, and *circ-IAA7* (Figure 7). Taken together, our results highlight the potential interplay between hormone metabolism and circRNA biogenesis, function, and degradation.

## Material and methods

### Plant materials and hormone treatments

After treatment with GA (100 μM), NAA (5 μM), and ddH_2_O for 4 h, 4-week-old whole seedlings of moso bamboo were collected and transferred to liquid nitrogen. Total RNA was isolated with the RNAprep Pure Plant Kit (Catalog No. DP441, Tiangen, Beijing, China), and concentrations and quality were determined using 2% agarose gel analysis and NanoDrop (Catalog No. 840-317400, ThermoFisher Scientific, Waltham, MA) quantification before libraries were constructed and high-throughput sequencing was performed.

### Library preparation and sequencing for circRNAs and linear RNAs

To ensure consistency and comparability of different omics, total RNA was divided into two tubes for circRNA and linear RNA sequencing. For circRNAs, rRNAs were depleted using the Ribo-Zero Magnetic Kit (Catalog No. MRZPL1224, Illumina, San Diego, CA), and then the samples were incubated with 3 U of RNase R (Catalog No. RNR07250, Epicentre, Madison, WI) for 15 min at 37 °C. The other group for conventional RNA-seq was incubated with oligo(T) magnetic beads (Catalog No. Dynabeads 61006, ThermoFisher Scientific, Meridian Rd) to enrich poly(A)+ RNAs. Subsequently, these RNAs were used for library preparation and subjected to sequencing with an Illumina HiSeq 2000 (Illumina).

### Bioinformatics analysis of circRNAs

For each sample, FASTQ reads were first filtered using the HTQC package (v1.92.1) [59] to remove low-quality reads using default parameters. Clean reads were then mapped to the reference genome [60] using TopHat (v2.0.11) [61]. Subsequently, the remaining unmapped reads were aligned to the genome using the TopHat-Fusion algorithm (v2.0.11) for identifying circRNAs with the CIRCexplorer2 annotation program [7]. Moreover, AS events were detected using CIRCexplorer2 *de novo* [7]. MICs were detected by overlapping exons following a previous study [23]. For evolutionary analysis, circRNAs in *A*. *thaliana* and *O*. *sativa* were also identified by the CIRCexplorer2 annotation program with the same parameters as moso bamboo using publicly available data [27], [28]. Reference sequences and annotation of *A*. *thaliana* (TAIR10) and *O*. *sativa* (MSU6.1) were obtained from The *Arabidopsis* Information Resource (TAIR) and the MSU Rice Genome Annotation Project Database, respectively. We used the bamboo circRNAs as queries against those of *A*. *thaliana* and *O*. *sativa* using BLASTN with an E-value of 0.01, and resulting hits sharing below 50% similar stretches of sequence were removed.

To compare circRNA expression among different samples, we first normalized circRNA abundance with RPM based on reads spanning the back-spliced junction to the total number of mapped reads (units in million). *P* values and false discovery rates (FDRs) were calculated using the DEGseq package [62]. Differentially expressed circRNAs were detected with fold change ≥ 2, *P* < 0.01, and FDR ≤ 0.01 as cutoff.

### Experimental validation of circRNA

For RNase R treatment and PCR amplification validation, we used the protocols from our previous study [63]. Briefly, 10× RNase R reaction buffer, RNase R, and diethyl pyrocarbonate (DEPC)-treated water were added and incubated for 15 min at 37 °C for RNase R digestion to take place before adding phenol–chloroform–isoamyl alcohol to stop the exonuclease digestion. The sample was then centrifuged at 13,000 *g* at 4 °C for 5 min, and the supernatant was transferred to a new 1.5-ml RNase-free tube containing LiCl, glycogen, and pre-chilled absolute ethanol (−20 °C) and finally inverted gently and stored at −80 °C for 1 h. Subsequently, RNase R-treated and control samples were reverse-transcribed to form complementary DNA (cDNA) for circRNA amplification with 40 cycles using divergent primers. PCR products with predicted sizes were dissected from a 2% agarose gel and directly sequenced. Divergent primers and convergent primers were designed to detect the candidate circRNA and positive control for linear RNA, and both are listed in Table S10.

### Functional annotation of moso bamboo

Core spliceosomal factor genes, RBP genes (including splicing factor genes), miRNA-related genes, translation-related genes, fast growth-related genes, and hormone-related genes were annotated using Blast2GO [64] with default options. GO enrichment analysis was performed by BiNGO [65]. The phylogenetic tree of QKI was prepared with ClustalX1.83 and Interactive Tree Of Life (iTQL) [66]. The KH domain of QKI was detected by the conserved domain database (CDD) of National Center of Biotechnology Information [67] and visualized by Gene Structure Display Server (GSDS) 2.0 [68].

### Protoplast isolation, plasmid construction, and transfection

Protoplasts of moso bamboo were isolated and transformed as previously described [69]. Briefly, shoots of seedlings were immediately transferred into a culture dish containing enzymatic solution and digested for 3 h at 25 °C with gentle shaking (50 r/min) in the dark. After adding an equal volume of CPW11M [consisting of 50-ml Cell and Protoplast Washing (CPW) buffer and 5.465 g mannitol] to a solution with a pH of 5.7, protoplast pellets were obtained from the miscible solution filtered through two layers of medical gauze and centrifuged for 3 min at 1200 r/min to remove the supernatant. After two additional washing steps using the CPW11M solution, the protoplast pellets were resuspended at a concentration of 1 × 10^5^–1 × 10^6^ protoplasts in 1 ml using MES-Mannitol-Mg (MMG) solution.

In this study, pUC22-35s-sGFP was used as the backbone for the construction of the overexpression and RNA interference (RNAi) vectors. To overexpress *circ-DCL4*, *circ-PKL*, *circ-CSLA1*, *circ-BRE1-1*/*circ-BRE1-2*, and the third linear exon of *NRT1*, the endogenous exons were cloned and flanked by 139-bp inverted complementary flanking introns using overlapping PCR [70]. The two inverted complementary exons (271 bp) from *PedQKI* (PH02Gene29161) were flanked across the intron of *Cunninghamia lanceolata* to construct the vector for *PedQKI* RNAi. *circ-NHLRC2* and *miR166* were amplified and flanked by the promoter (CaMV 35S) and nopaline synthase (NOS) terminator using overlapping PCR. Then, the two resulting products were inserted in the plasmid to enable the co-expression of both *circ-NHLRC2* and *miR166*.

The recombinant plasmids were transfected using the Polyethylene Glycol (PEG)-mediated method. For each sample, 100-μl plasmid, protoplast, and 110-μl PEG solution were mixed and incubated at 25 °C. After incubation, the mixture was added to 5-ml CPW11M solution and centrifuged for 3 min at 1200 r/min. The remaining protoplasts without supernatant were resuspended with 10-ml CPW11M and centrifuged again at 1200 r/min for 3 min. Finally, after adding 10-ml CPW11M solution, the transfected protoplasts were incubated at 25 °C in the dark for 12–20 h. The expression of transcripts including circRNAs, mRNAs, and pre-miRNAs was detected by semi-quantitative RT-PCR using divergent or convergent primers (Table S10).

### Identification of AS events from RNA-seq

Low-quality reads were cleaned with default parameters using the HTQC package (v1.92.1) [59], and the remaining reads were mapped to the genome [60] by TopHat (v2.0.11) [61]. Aligned reads were used to assemble transcriptome annotation applying Cufflinks (v2.1.1) with default parameters [71]. Differential expression AS events were detected by rMATS.3.2.2 with the following option “-a 8 -c 0.0001 - analysis U” [72]. The parameters for “-a” and “-c” represented anchor length and the cutoff splicing difference, respectively. The default anchor length was 8 for RNA-seq splice-aware alignment. The default cutoff splicing difference was 0.0001 for 0.01% difference. To determine whether the frequency of AS events located in the transcribed region of circRNAs was considerably higher than other regions of linear RNA, we randomly selected the same number of transcribed regions without generating circRNAs, and the number of sequences affected by AS was calculated. After repeating the process 1000 times, we determined the mean value and the standard deviation of the simulation data. PCCs between ecircRNAs and four types of AS events were calculated using the expression profiles of ecircRNAs and AS events, which was represented by back-splicing junction reads for ecircRNAs and splicing junction for AS events, respectively. In addition, PCCs between circRNA and linear RNA were calculated based on expression of circRNAs from circRNA sequencing and expression of linear RNA from mRNA sequencing.

### Construction of customized degradome sequencing libraries

The decay of circRNAs lacking the 3′ poly(A) tail of circRNAs cannot be detected by conventional degradome sequencing. A modified degradome library preparation was developed based on 5′ rapid-amplification of cDNA ends (RACE) library preparation. The Dynabeads mRNA Purification Kit (Catalog No. 61006, ThermoFisher Scientific, Carlsbad, CA) was used according to the manufacturer’s instructions to extract total RNA from 4-week-old bamboo seedlings treated with GA, NAA, and H_2_O. Total RNA was then separated into poly(A)− and poly(A)+ groups. Subsequently, rRNAs were removed from the poly(A)− group using the Ribo-Zero Magnetic Kit (Catalog No. MRZPL1224, Illumina). The free 5′ monophosphates of the cleavage transcripts of poly(A)− and poly(A)+ RNA were both ligated to the 5′ RNA adapter. Following reverse transcription with biotinylated random primers, the cDNA library was sequenced using an Illumina HiSeq 2000.

### Bioinformatics analysis for customized degradome sequencing

For customized degradome sequencing, we developed a computational strategy to detect and compare the accumulated decay events termed as degradome peaks with candidate miRNA-mediated cleavage sites in circRNAs. First, degradome sequencing reads were aligned to the genome using Bowtie 2 (v2.2.1) with default parameters [36]. The mapping reads were calculated at the 5′ end alignment positions and counted as 1/*n* to determine the distribution of reads and the degradome peaks, in which *n* is the total number of mapped reads. The degradome peak was selected by the following cutoff: the percent abundance of cleavage sites was 50% or more in each continuous 21 nt consisting of the 10 nt upstream and downstream of cleavage sites. The *P* value cutoff of cleavage sites calculated by binomial test was determined as 0.05. Then, the 25 upstream and downstream nucleotides of cleavage sites were aligned to mature miRNAs by RNAplex [37] to identify the target RNA, in which the minimum free energy (MFE) ratio was set as 0.7 or less and the sliced sites were in the ±1 region around cleavage sites. To further detect cleavage sites spanning back-splicing sites, we first extracted the 50 upstream and downstream nucleotides of back-splicing sites from circRNAs including degradome peaks. Then, all reads from the poly(A)− library were aligned to back-splicing junction regions of circRNAs using Bowtie 2 with default parameters [36]. Finally, the cleavage of circRNAs supported by degradome reads was identified.

### Small RNA sequencing and bioinformatics analysis

To rapidly and effectively obtain high-quality small RNAs for mock and hormone-treated samples, PEG8000 precipitation was utilized to isolate the small RNAs from 5 μg of total RNA for each library and then the 3′ adapter and 5′ adapter were successively ligated to small RNAs before reverse transcription. Finally, the DNA product was enriched using 3.5% polyacrylamide gel electrophoresis (PAGE), and bands of approximately 200 bp were isolated before sequencing using HiSeq 2500.

The raw RNA reads were processed to remove 5′ adapters and 3′ adapters using the fastx-clipper function of the FASTX-Toolkit. The filtered reads were subjected to alignment against the Rfam database [73] using Bowtie 2 (v2.2.1) [36] to remove the common RNA families including rRNAs, transfer RNAs (tRNAs), small nuclear RNAs (snRNAs), and small nucleolar RNAs (snoRNAs). The remaining sequences were aligned against the miRBase database (v21) [74] using the BLAST algorithm to identify miRNAs, allowing for an E-value < 0.05 and three mismatches in total between targets reads and known miRNAs. Completely matched sequences were deemed as conserved miRNAs, and other sequences with no more than three mismatches or gaps were considered as variant miRNAs. RPM mapped reads were used to normalize the expression of miRNA from different libraries.

### Identification and annotation of ORFs from circRNAs

To predict and annotate the cORFs, circRNA sequences excluding introns were multiplied four times for ORF prediction (Figure S5) applying TransDecoder with lowest length > 10 aa [75]. Subsequently, three protein databases including Nr [76], UniProt, and PsORF [32] were searched using BLASTP to detect their homologous proteins with known function using the following parameters: identity > 80%, E-value < 0.01, and the length of alignment > 50%. By the same measurement, uORFs and dORFs derived from linear RNAs were predicted and annotated. To detect unique peptides for each of the ORFs in cORFs, uORFs, dORFs, and pORFs, we obtained raw proteome data based on label-free and tandem mass tags approaches [77], [78]. We then performed four independent searches to identify the peptide-matching ORFs using MaxQuant with standard parameters [79]. Subsequently, we removed the peptides matching more than one ORF, and the ORFs with at least one unique peptide were retained.

### Transformation procedure for six circRNAs

The pCAMBIA1390 vector including six circRNAs and flanking inverted complementary intron sequences were transformed into Kitaake (*O*. *sativa* ssp *japonica*). The mature embryos were used as the material for callus induction and *Agrobacterium*-mediated transformation. The hygromycin-resistant callus was transferred into differentiation medium for regeneration. To validate transformation, genomic DNA from rice leaves was extracted using cetyl trimethylammonium bromide (CTAB) methods, and the expected fragments were amplified using primers of hygromycin genes. Expression levels of the six circRNA transcripts were further detected by RT-PCR using divergent primers (Table S10). All seeds from wild-type and T1 generation plants overexpressing circRNAs were incubated in a petri dish to ensure consistent germination. Germinated seeds were transferred to nutrient soil for seedling growing. Then, plant heights were measured as the height from soil surface to the top leaf. In total, 20 seedlings were selected for each overexpressed circRNA for calculation of above-ground plant height.

## Code availability

The codes have been submitted to BioCode at the National Genomics Data Center (NGDC), Beijing Institute of Genomics (BIG), Chinese Academy of Sciences (CAS) / China National Center for Bioinformation (CNCB) (BioCode: BT007322), which are publicly accessible at https://ngdc.cncb.ac.cn/biocode/tools/BT007322.

## Data availability

Raw sequencing data from mRNA sequencing, circRNA sequencing, degradome sequencing, and small RNA sequencing have been deposited in the Genome Sequence Archive [80] at the NGDC, BIG, CAS / CNCB (GSA: CRA007877) , and are publicly accessible at https://ngdc.cncb.ac.cn/gsa. Raw sequencing data for all the libraries have also been submitted to the NCBI Gene Expression Omnibus (GEO: PRJNA707140).

## Competing interests

The authors have declared no competing interests.

## CRediT authorship contribution statement

**Yongsheng Wang:** Methodology, Investigation, Software, Validation, Writing – original draft. **Huihui Wang:** Investigation, Validation. **Huiyuan Wang:** Software, Data curation. **Ruifan Zhou:** Investigation, Validation. **Ji Wu:** Validation. **Zekun Zhang:** Software. **Yandong Jin:** Validation. **Tao Li:** Validation. **Markus V. Kohnen:** Software. **Xuqing Liu:** Validation. **Wentao Wei:** Validation. **Kai Chen:** Validation. **Yubang Gao:** Software. **Jiazhi Ding:** Validation. **Hangxiao Zhang:** Software. **Bo Liu:** Conceptualization. **Chentao Lin:** Conceptualization. **Lianfeng Gu:** Conceptualization, Writing – review & editing, Supervision. All authors have read and approved the final manuscript.

## Supplementary material

Supplementary Figure S1 **GO enrichment analysis for the homologous circRNAs detected in three plants** GO terms on the X-axis are categorized into three types with different colors. The Y-axis indicates the percentage (left Y-axis) and number (right Y-axis) of host genes including homologous circRNAs.

Supplementary Figure S2 **Length of flanking introns** Cumulative curve and boxplots of the length of upstream and downstream flanking introns of circRNAs in comparison with control introns.

Supplementary Figure S3 **Distribution of PCCs between circRNAs and RBPs A.** Bar plot of the distribution of PCCs between circRNAs and 24 core spliceosomal components, 92 splicing factors, and 1132 other RBPs excluding splicing factors and core spliceosomal components in bamboo. **B.** Scatterplot of distribution of PCCs between circRNAs and seven RPBs (SF3A, SF3B, NF90, DHX9, FUS, HNRNPL, and QKI) in bamboo.

Supplementary Figure S4 **Evolutionary trees of human QKI and its 34 homologous proteins in moso bamboo** KH domains are indicated as yellow bars. KH, K homology.

Supplementary Figure S5 **Translatable circRNAs in moso bamboo A.** Overview of the identification and annotation of the potential cORFs. **B.** The histogram plot shows the percentage of homologous cORFs, uORFs, and dORFs with known protein databases. **C.** Phylogenetic analysis of the cORF of *circ-GLO5* and six homologous cORFs from other species. **D.** Number of the translatable cORFs, uORFs, dORFs, and pORFs based on proteomics. **E.** The MS spectra of cORFs from *circ-P4H-1*. The a, b, and y ions are indicated by red, green, and blue lines, respectively. ORFs, open reading frames; cORFs, ORFs of circRNAs; uORFs, upstream ORFs; dORFs, downstream ORFs; pORFs, primary ORFs; MS, mass spectrometry/mass spectrometry ; m/z, Mass to charge ratio ; Nr, Non-Redundant Protein Sequence Database; PsORF, Database Of Plant Small ORFs; UniProt, Universal Protein Database.

Supplementary Figure S6 **Homology analysis of RNase L A.** Multiple sequence alignment of RNase L in different species. **B.** The histogram plot shows the number of homologous proteins.

Supplementary Figure S7 **Construction and analysis of degradome libraries A.** The histogram plot shows the number of miRNAs and top 15 miRNA families. **B.** The boxplot shows the distribution of reads in the 5' UTR, CDSs, and 3' UTR from poly(A)+ and poly(A)− degradome libraries. **C.** A computational pipeline for identifying cleavage sites based on two types of degradome libraries. **D.** A computational pipeline for detecting decaying transcripts of circRNAs spanning back-splicing sites.

Supplementary Figure S8 **Potential function of hormone-induced circRNAs A.** Heatmap showing expression levels of circular transcripts related to the GO biological process terms plant hormones, second messenger, cell inclusion bodies, and plant organs. **B.** The events in the presence of gibberellin. **C.** The events in the presence of auxin. Red genes indicate host genes that generated circRNAs. The light-green box indicates biosynthesis, light purple indicates transport, and light yellow indicates signaling. Black arrows indicate positive effects, and black dashed lines indicate negative effects.

Supplementary Figure S9 **The overexpression of six candidate circular RNAs (*circ-SPY*, *circ-MYBS3*, *circ-WRKY4*, *circ-CslA1*, *circ-AGO1A*, and *circ-GID1*) in rice.**

Supplementary Table S1 **Statistical data for sequencing and circRNAs in seedlings of moso bamboo**

Supplementary Table S2 **Core spliceosomal factors, splicing factors, and other RBPs with higher Pearson correlation coefficients**

Supplementary Table S3 **Alternative splicing events located in transcribed regions of circRNAs in the three species**

Supplementary Table S4 **Identified mature miRNAs and miRNA-related gene in moso bamboo**

Supplementary Table S5 **CircRNAs and linear RNAs decayed by miRNAs**

Supplementary Table S6 **Translatable circRNAs and translation-related proteins**

Supplementary Table S7 **Differentially expressed circRNAs and GA and NAA hormone-related genes**

Supplementary Table S8 **Genes from bamboo transformed into *Arabidopsis thaliana*, *Oryza sativa*, and *Nicotiana tabacum***

Supplementary Table S9 **Interplay between hormone (GA and NAA) treatment and circRNA metabolism**

Supplementary Table S10 **Primers for validation and transformation of circRNAs**

## References

[1] Sanger H.L., Klotz G., Riesner D., Gross H.J., Kleinschmidt A.K. (1976). Viroids are single-stranded covalently closed circular RNA molecules existing as highly base-paired rod-like structures. Proc Natl Acad Sci U S A.

[2] Kos A., Dijkema R., Arnberg A.C., van der Meide P.H., Schellekens H. (1986). The hepatitis delta (δ) virus possesses a circular RNA. Nature.

[3] Zhang X.O., Wang H.B., Zhang Y., Lu X., Chen L.L., Yang L. (2014). Complementary sequence-mediated exon circularization. Cell.

[4] Zhang Y., Zhang X.O., Chen T., Xiang J.F., Yin Q.F., Xing Y.H. (2013). Circular intronic long noncoding RNAs. Mol Cell.

[5] Capel B., Swain A., Nicolis S., Hacker A., Walter M., Koopman P. (1993). Circular transcripts of the testis-determining gene *Sry* in adult mouse testis. Cell.

[6] Gao Y., Wang J., Zheng Y., Zhang J., Chen S., Zhao F. (2016). Comprehensive identification of internal structure and alternative splicing events in circular RNAs. Nat Commun.

[7] Zhang X.O., Dong R., Zhang Y., Zhang J.L., Luo Z., Zhang J. (2016). Diverse alternative back-splicing and alternative splicing landscape of circular RNAs. Genome Res.

[8] Ivanov A., Memczak S., Wyler E., Torti F., Porath H.T., Orejuela M.R. (2015). Analysis of intron sequences reveals hallmarks of circular RNA biogenesis in animals. Cell Rep.

[9] Liang D., Wilusz J.E. (2014). Short intronic repeat sequences facilitate circular RNA production. Genes Dev.

[10] Westholm J.O., Miura P., Olson S., Shenker S., Joseph B., Sanfilippo P. (2014). Genome-wide analysis of *Drosophila* circular RNAs reveals their structural and sequence properties and age-dependent neural accumulation. Cell Rep.

[11] Lu T., Cui L., Zhou Y., Zhu C., Fan D., Gong H. (2015). Transcriptome-wide investigation of circular RNAs in rice. RNA.

[12] Liang D., Tatomer D.C., Luo Z., Wu H., Yang L., Chen L.L. (2017). The output of protein-coding genes shifts to circular RNAs when the pre-mRNA processing machinery is limiting. Mol Cell.

[13] Conn S.J., Pillman K.A., Toubia J., Conn V.M., Salmanidis M., Phillips C.A. (2015). The RNA binding protein quaking regulates formation of circRNAs. Cell.

[14] Rybak-Wolf A., Stottmeister C., Glazar P., Jens M., Pino N., Giusti S. (2015). Circular RNAs in the mammalian brain are highly abundant, conserved, and dynamically expressed. Mol Cell.

[15] Aktas T., Ilik I.A., Maticzka D., Bhardwaj V., Rodrigues C.P., Mittler G. (2017). DHX9 suppresses RNA processing defects originating from the *Alu* invasion of the human genome. Nature.

[16] Li X., Liu C.X., Xue W., Zhang Y., Jiang S., Yin Q.F. (2017). Coordinated circRNA biogenesis and function with NF90/NF110 in viral infection. Mol Cell.

[17] Fei T., Chen Y., Xiao T., Li W., Cato L., Zhang P. (2017). Genome-wide CRISPR screen identifies HNRNPL as a prostate cancer dependency regulating RNA splicing. Proc Natl Acad Sci U S A.

[18] Hansen T.B., Wiklund E.D., Bramsen J.B., Villadsen S.B., Statham A.L., Clark S.J. (2011). miRNA-dependent gene silencing involving Ago2-mediated cleavage of a circular antisense RNA. EMBO J.

[19] Li Z., Huang C., Bao C., Chen L., Lin M., Wang X. (2015). Exon-intron circular RNAs regulate transcription in the nucleus. Nat Struct Mol Biol.

[20] Conn V.M., Hugouvieux V., Nayak A., Conos S.A., Capovilla G., Cildir G. (2017). A circRNA from *SEPALLATA3* regulates splicing of its cognate mRNA through R-loop formation. Nat Plants.

[21] Chu Q., Zhang X., Zhu X., Liu C., Mao L., Ye C. (2017). PlantcircBase: a database for plant circular RNAs. Mol Plant.

[22] Enns L.C., Kanaoka M.M., Torii K.U., Comai L., Okada K., Cleland R.E. (2005). Two callose synthases, GSL1 and GSL5, play an essential and redundant role in plant and pollen development and in fertility. Plant Mol Biol.

[23] Wang Y., Gao Y., Zhang H., Wang H., Liu X., Xu X. (2019). Genome-wide profiling of circular RNAs in the rapidly growing shoots of moso bamboo (*Phyllostachys edulis*). Plant Cell Physiol.

[24] Salzman J., Chen R.E., Olsen M.N., Wang P.L., Brown P.O. (2013). Cell-type specific features of circular RNA expression. PLoS Genet.

[25] Starke S., Jost I., Rossbach O., Schneider T., Schreiner S., Hung L.H. (2015). Exon circularization requires canonical splice signals. Cell Rep.

[26] Liu X., Gao Y., Liao J., Miao M., Chen K., Xi F. (2021). Genome-wide profiling of circular RNAs, alternative splicing, and R-loops in stem-differentiating xylem of *Populus trichocarpa*. J Integr Plant Biol.

[27] Secco D., Jabnoune M., Walker H., Shou H., Wu P., Poirier Y. (2013). Spatio-temporal transcript profiling of rice roots and shoots in response to phosphate starvation and recovery. Plant Cell.

[28] Li Z., Wang S., Cheng J., Su C., Zhong S., Liu Q. (2016). Intron lariat RNA inhibits microRNA biogenesis by sequestering the dicing complex in *Arabidopsis*. PLoS Genet.

[29] Xu W., Xu H., Li K., Fan Y., Liu Y., Yang X. (2017). The R-loop is a common chromatin feature of the *Arabidopsis* genome. Nat Plants.

[30] Shafiq S., Chen C., Yang J., Cheng L., Ma F., Widemann E. (2017). DNA topoisomerase 1 prevents R-loop accumulation to modulate auxin-regulated root development in rice. Mol Plant.

[31] Chen Y., Yang F., Fang E., Xiao W., Mei H., Li H. (2019). Circular RNA *circAGO2* drives cancer progression through facilitating HuR-repressed functions of AGO2-miRNA complexes. Cell Death Differ.

[32] Chen Y., Li D., Fan W., Zheng X., Zhou Y., Ye H. (2020). PsORF: a database of small ORFs in plants. Plant Biotechnol J.

[33] Liu C.X., Li X., Nan F., Jiang S., Gao X., Guo S.K. (2019). Structure and degradation of circular RNAs regulate PKR activation in innate immunity. Cell.

[34] German M.A., Pillay M., Jeong D.H., Hetawal A., Luo S., Janardhanan P. (2008). Global identification of microRNA-target RNA pairs by parallel analysis of RNA ends. Nat Biotechnol.

[35] Addo-Quaye C., Eshoo T.W., Bartel D.P., Axtell M.J. (2008). Endogenous siRNA and miRNA targets identified by sequencing of the *Arabidopsis* degradome. Curr Biol.

[36] Langmead B., Salzberg S.L. (2012). Fast gapped-read alignment with Bowtie 2. Nat Methods.

[37] Tafer H., Hofacker I.L. (2008). RNAplex: a fast tool for RNA–RNA interaction search. Bioinformatics.

[38] Wang W., Gu L., Ye S., Zhang H., Cai C., Xiang M. (2017). Genome-wide analysis and transcriptomic profiling of the auxin biosynthesis, transport and signaling family genes in moso bamboo (*Phyllostachys heterocycla*). BMC Genomics.

[39] Zhang H., Wang H., Zhu Q., Gao Y., Wang H., Zhao L. (2018). Transcriptome characterization of moso bamboo (*Phyllostachys edulis*) seedlings in response to exogenous gibberellin applications. BMC Plant Biol.

[40] Zhao Y. (2008). The role of local biosynthesis of auxin and cytokinin in plant development. Curr Opin Plant Biol.

[41] Binenbaum J., Weinstain R., Shani E. (2018). Gibberellin localization and transport in plants. Trends Plant Sci.

[42] Liepman A.H., Nairn C.J., Willats W.G.T., Sorensen I., Roberts A.W., Keegstra K. (2007). Functional genomic analysis supports conservation of function among cellulose synthase-like a gene family members and suggests diverse roles of mannans in plants. Plant Physiol.

[43] Hou D., Li L., Ma T., Pei J., Zhao Z., Lu M. (2021). The *SOC1*-like gene *BoMADS50* is associated with the flowering of *Bambusa oldhamii*. Hortic Res.

[44] Zhang Y., Gu L., Hou Y., Wang L., Deng X., Hang R. (2015). Integrative genome-wide analysis reveals HLP1, a novel RNA-binding protein, regulates plant flowering by targeting alternative polyadenylation. Cell Res.

[45] Mi S., Cai T., Hu Y., Chen Y., Hodges E., Ni F. (2008). Sorting of small RNAs into *Arabidopsis* argonaute complexes is directed by the 5′ terminal nucleotide. Cell.

[46] Hou D., Cheng Z., Xie L., Li X., Li J., Mu S. (2018). The R2R3MYB gene family in *Phyllostachys edulis*: genome-wide analysis and identification of stress or development-related *R2R3MYBs*. Front Plant Sci.

[47] Guo Z., Zhang Z., Yang X., Yin K., Chen Y., Zhang Z. (2020). *PSBR1*, encoding a mitochondrial protein, is regulated by brassinosteroid in moso bamboo (*Phyllostachys edulis*). Plant Mol Biol.

[48] Cui X., Zhang Y., Qi F., Gao J., Chen Y., Zhang C. (2013). Overexpression of a moso bamboo (*Phyllostachys edulis*) transcription factor gene *PheWRKY1* enhances disease resistance in transgenic *Arabidopsis thaliana*. Botany.

[49] Liu J., Cheng Z., Xie L., Li X., Gao J. (2019). Multifaceted role of *PheDof12-1* in the regulation of flowering time and abiotic stress responses in moso bamboo (*Phyllostachys edulis*). Int J Mol Sci.

[50] Wu M., Liu H., Han G., Cai R., Pan F., Xiang Y. (2017). A moso bamboo *WRKY* gene *PeWRKY83* confers salinity tolerance in transgenic *Arabidopsis* plants. Sci Rep.

[51] Cheng X., Wang Y., Xiong R., Gao Y., Yan H., Xiang Y. (2020). A moso bamboo gene *VQ28* confers salt tolerance to transgenic *Arabidopsis* plants. Planta.

[52] Wan T., Li Q., Lou S., Yang Y., Peng L., Lin Z. (2019). GSK3/shaggy-like kinase 1 ubiquitously regulates cell growth from *Arabidopsis* to moso bamboo (*Phyllostachys edulis*). Plant Sci.

[53] Zhang Y., Tang D., Lin X., Ding M., Tong Z. (2018). Genome-wide identification of MADS-box family genes in moso bamboo (*Phyllostachys edulis*) and a functional analysis of *PeMADS5* in flowering. BMC Plant Biol.

[54] Wang T., Yang Y., Lou S., Wei W., Zhao Z., Ren Y. (2020). Genome-wide characterization and gene expression analyses of GATA transcription factors in moso bamboo (*Phyllostachys edulis*). Int J Mol Sci.

[55] Wang L., Zhao H., Chen D., Li L., Sun H., Lou Y. (2016). Characterization and primary functional analysis of a bamboo *NAC* gene targeted by miR164b. Plant Cell Rep.

[56] Li L., Mu S., Cheng Z., Cheng Y., Zhang Y., Miao Y. (2017). Characterization and expression analysis of the WRKY gene family in moso bamboo. Sci Rep.

[57] Sun H., Li L., Lou Y., Zhao H., Yang Y., Wang S. (2017). The bamboo aquaporin gene *PeTIP4;1–1* confers drought and salinity tolerance in transgenic *Arabidopsis*. Plant Cell Rep.

[58] Guo X., Chen H., Liu Y., Chen W., Ying Y., Han J. (2020). The acid invertase gene family is involved in internode elongation in *Phyllostachys heterocycla* cv. *pubescens*. Tree Physiol.

[59] Yang X., Liu D., Liu F., Wu J., Zou J., Xiao X. (2013). HTQC: a fast quality control toolkit for Illumina sequencing data. BMC Bioinformatics.

[60] Zhao H., Gao Z., Wang L., Wang J., Wang S., Fei B. (2018). Chromosome-level reference genome and alternative splicing atlas of moso bamboo (*Phyllostachys edulis*). Gigascience.

[61] Trapnell C., Pachter L., Salzberg S.L. (2009). TopHat: discovering splice junctions with RNA-seq. Bioinformatics.

[62] Wang L., Feng Z., Wang X., Wang X., Zhang X. (2010). DEGseq: an R package for identifying differentially expressed genes from RNA-seq data. Bioinformatics.

[63] Wang Y., Wang H., Xi F., Wang H., Han X., Wei W. (2020). Profiling of circular RNA *N*^6^-methyladenosine in moso bamboo (*Phyllostachys edulis*) using nanopore-based direct RNA sequencing. J Integr Plant Biol.

[64] Conesa A., Götz S., García-Gómez J.M., Terol J., Talón M., Robles M. (2005). Blast2GO: a universal tool for annotation, visualization and analysis in functional genomics research. Bioinformatics.

[65] Maere S., Heymans K., Kuiper M. (2005). BiNGO: a Cytoscape plugin to assess overrepresentation of Gene Ontology categories in biological networks. Bioinformatics.

[66] Letunic I., Bork P. (2019). Interactive Tree Of Life (iTOL) v4: recent updates and new developments. Nucleic Acids Res.

[67] Marchler-Bauer A., Derbyshire M.K., Gonzales N.R., Lu S., Chitsaz F., Geer L.Y. (2015). CDD: NCBI’s conserved domain database. Nucleic Acids Res.

[68] Hu B., Jin J., Guo A.Y., Zhang H., Luo J., Gao G. (2015). GSDS 2.0: an upgraded gene feature visualization server. Bioinformatics.

[69] Chen K., Hu K., Xi F., Wang H., Kohnen M.V., Gao P. (2021). High-efficient and transient transformation of moso bamboo (*Phyllostachys edulis*) and ma bamboo (*Dendrocalamus latiflorus* Munro). J Plant Biol.

[70] Bryksin A.V., Matsumura I. (2010). Overlap extension PCR cloning: a simple and reliable way to create recombinant plasmids. Biotechniques.

[71] Trapnell C., Roberts A., Goff L., Pertea G., Kim D., Kelley D.R. (2012). Differential gene and transcript expression analysis of RNA-seq experiments with TopHat and Cufflinks. Nat Protoc.

[72] Shen S., Park J., Lu Z.X., Lin L., Henry M.D., Wu Y. (2014). rMATS: robust and flexible detection of differential alternative splicing from replicate RNA-seq data. Proc Natl Acad Sci U S A.

[73] Griffiths-Jones S., Bateman A., Marshall M., Khanna A., Eddy S.R. (2003). Rfam: an RNA family database. Nucleic Acids Res.

[74] Kozomara A., Griffiths-Jones S. (2013). miRBase: annotating high confidence microRNAs using deep sequencing data. Nucleic Acids Res.

[75] Haas B.J., Papanicolaou A., Yassour M., Grabherr M., Blood P.D., Bowden J. (2013). *De novo* transcript sequence reconstruction from RNA-seq using the Trinity platform for reference generation and analysis. Nat Protoc.

[76] Pruitt K.D., Tatusova T., Maglott D.R. (2007). NCBI reference sequences (RefSeq): a curated non-redundant sequence database of genomes, transcripts and proteins. Nucleic Acids Res.

[77] Yu X., Wang Y., Kohnen M.V., Piao M., Tu M., Gao Y. (2019). Large scale profiling of protein isoforms using label-free quantitative proteomics revealed the regulation of nonsense-mediated decay in moso bamboo (*Phyllostachys edulis*). Cells.

[78] Tao G.Y., Ramakrishnan M., Vinod K.K., Yrjala K., Satheesh V., Cho J. (2020). Multi-omics analysis of cellular pathways involved in different rapid growth stages of moso bamboo. Tree Physiol.

[79] Tyanova S., Temu T., Cox J. (2016). The MaxQuant computational platform for mass spectrometry-based shotgun proteomics. Nat Protoc.

[80] Chen T., Chen X., Zhang S., Zhu J., Tang B., Wang A. (2021). The Genome Sequence Archive Family: toward explosive data growth and diverse data types. Genomics Proteomics Bioinformatics.

