# Supplementary figures and images for: Multi-omics of Circular RNAs and Their Responses to Hormones in Moso Bamboo (Phyllostachys edulis)
Source: Genomics Proteomics Bioinformatics. 2023 Feb 16;21(4):866–85. doi: 10.1016/j.gpb.2023.01.007 (PMC10787125; doi:10.1016/j.gpb.2023.01.007)

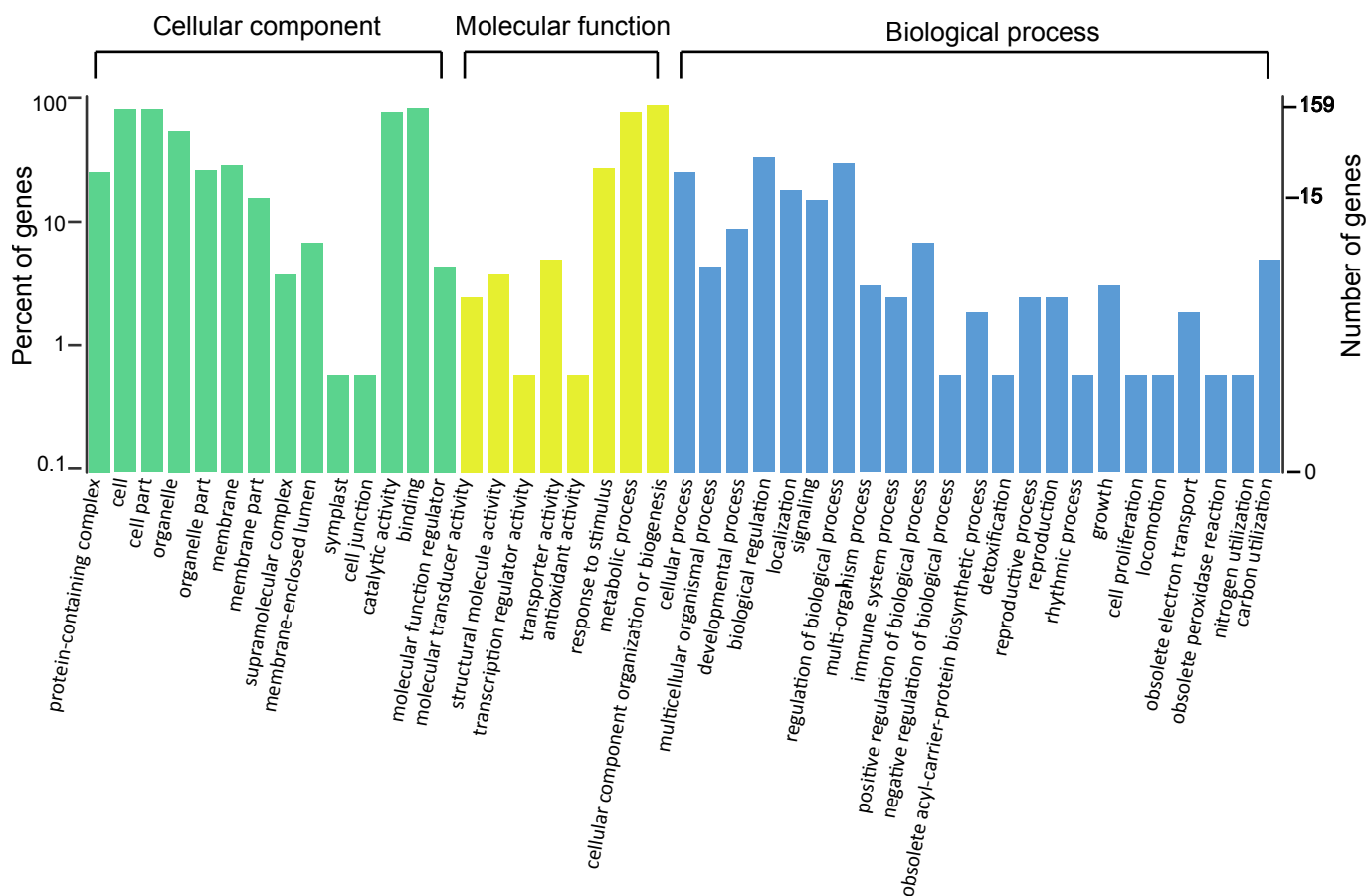

Supplement: Supplementary Figure S1 — GO enrichment analysis for the homologous circRNAs detected in three plants GO terms on the X-axis are categorized into three types with different colors. The Y-axis indicates the percentage (left Y-axis) and number (right Y-axis) of host genes including homologous circRNAs. [file mmc1.pdf]

Exon circRNA Intron Flanking intron

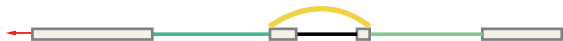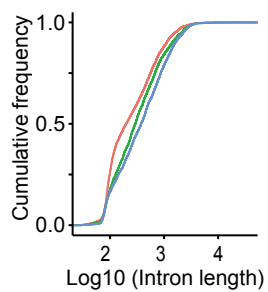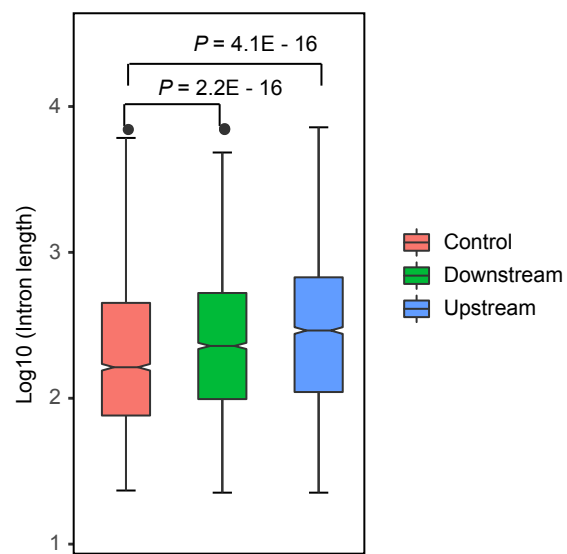

Supplement: Supplementary Figure S2 — Length of flanking introns Cumulative curve and boxplots of the length of upstream and downstream flanking introns of circRNAs in comparison with control introns. [file mmc2.pdf]

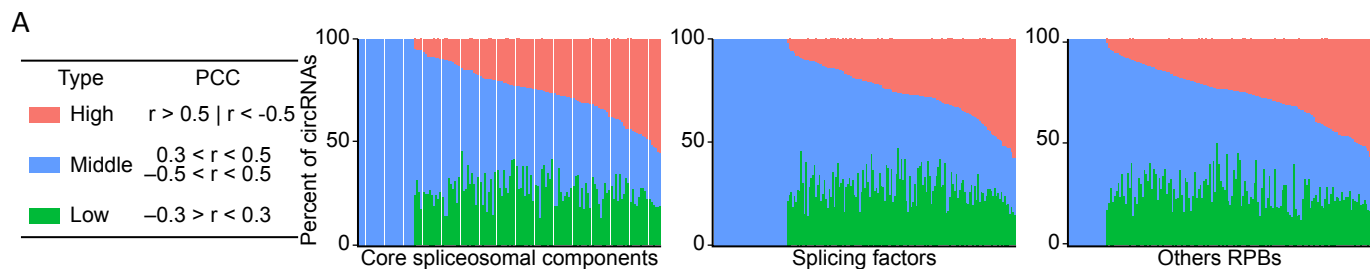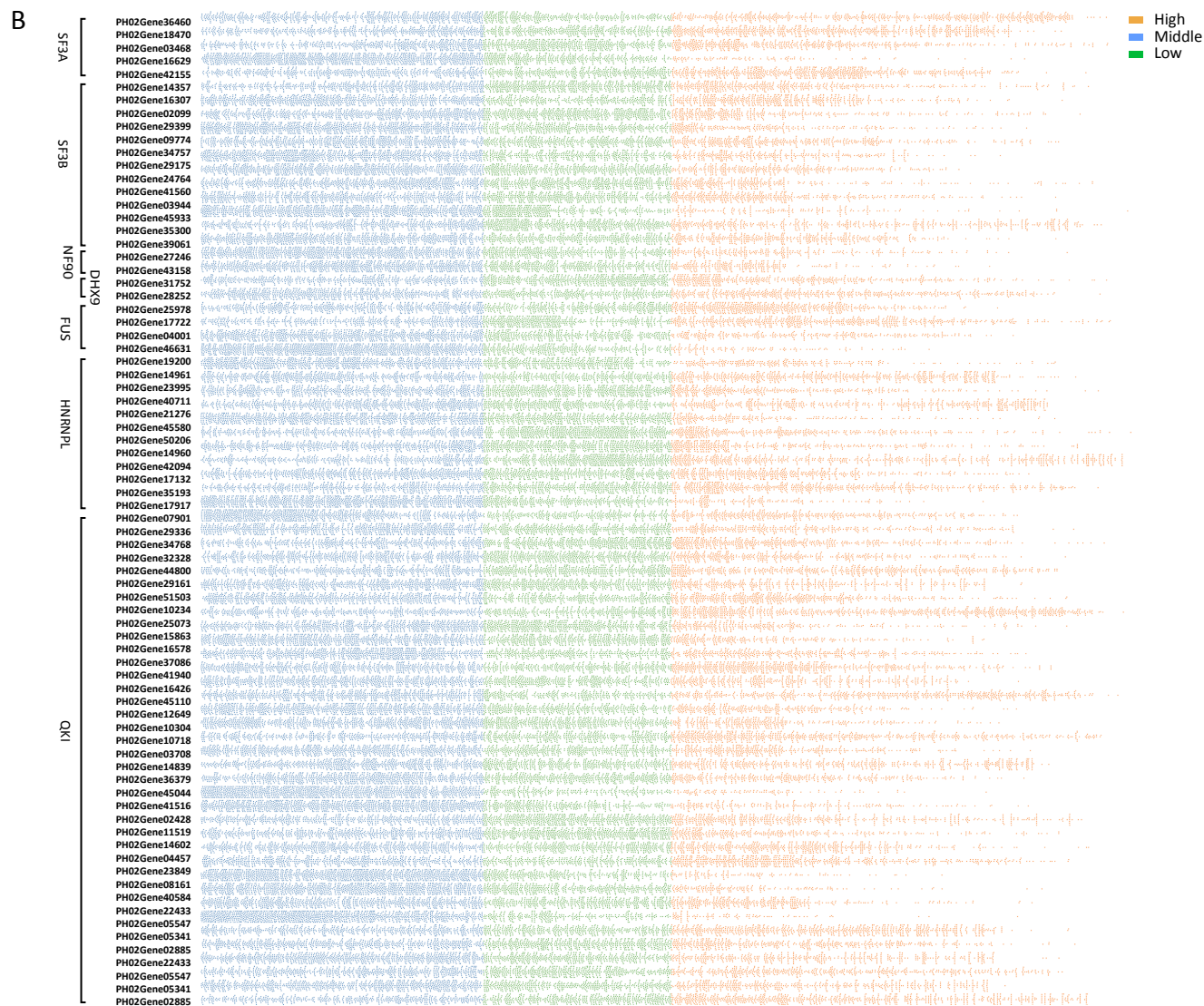

Supplement: Supplementary Figure S3 — Distribution of PCCs between circRNAs and RBPs A. Bar plot of the distribution of PCCs between circRNAs and 24 core spliceosomal components, 92 splicing factors, and 1132 other RBPs excluding splicing factors and core spliceosomal components in bamboo. B. Scatterplot of distribution of PCCs between circRNAs and seven RPBs (SF3A, SF3B, NF90, DHX9, FUS, HNRNPL, and QKI) in bamboo. [file mmc3.pdf]

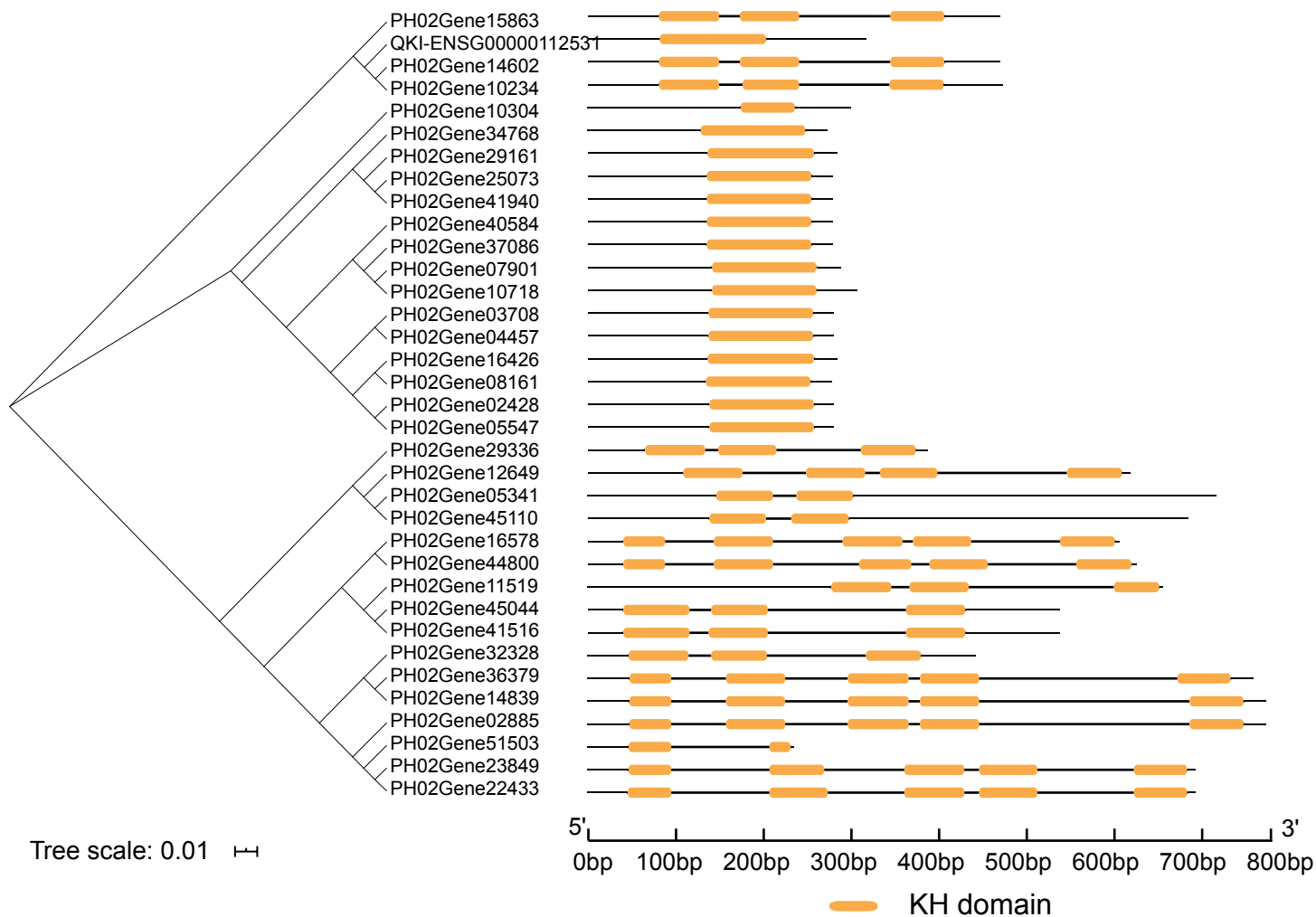

Supplement: Supplementary Figure S4 — Evolutionary trees of human QKI and its 34 homologous proteins in moso bamboo KH domains are indicated as yellow bars. KH, K homology. [file mmc4.pdf]

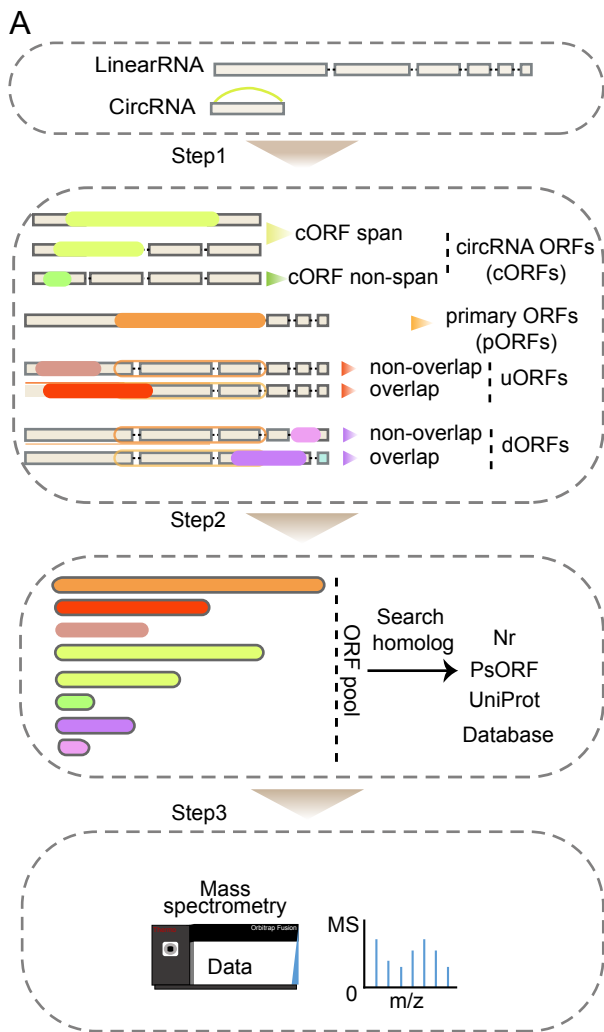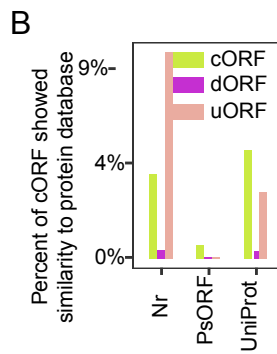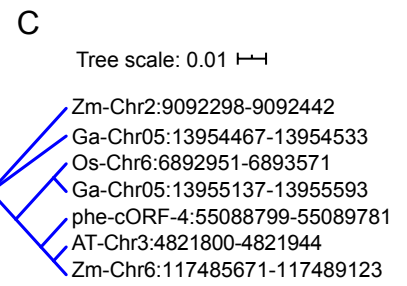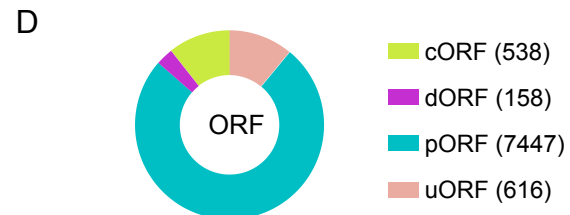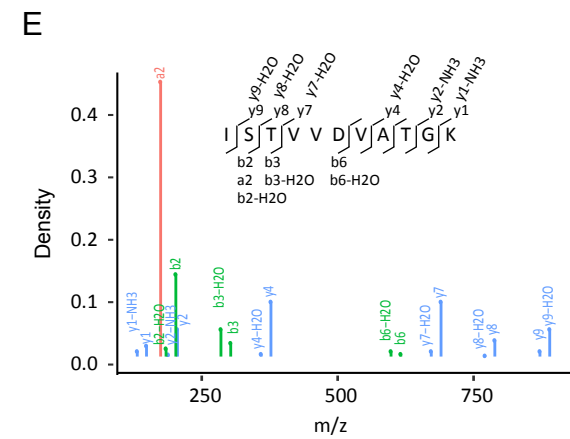

Supplement: Supplementary Figure S5 — Translatable circRNAs in moso bamboo A. Overview of the identification and annotation of the potential cORFs. B. The histogram plot shows the percentage of homologous cORFs, uORFs, and dORFs with known protein databases. C. Phylogenetic analysis of the cORF of circ-GLO5 and six homologous cORFs from other species. D. Number of the translatable cORFs, uORFs, dORFs, and pORFs based on proteomics. E. The MS spectra of cORFs from circ-P4H-1. The a, b, and y ions are indicated by red, green, and blue lines, respectively. ORFs, open reading frames; cORFs, ORFs of circRNAs; uORFs, upstream ORFs; dORFs, downstream ORFs; pORFs, primary ORFs; MS, mass spectrometry/mass spectrometry ; m/z, Mass to charge ratio ; Nr, Non-Redundant Protein Sequence Database; PsORF, Database Of Plant Small ORFs; UniProt, Universal Protein Database. [file mmc5.pdf]

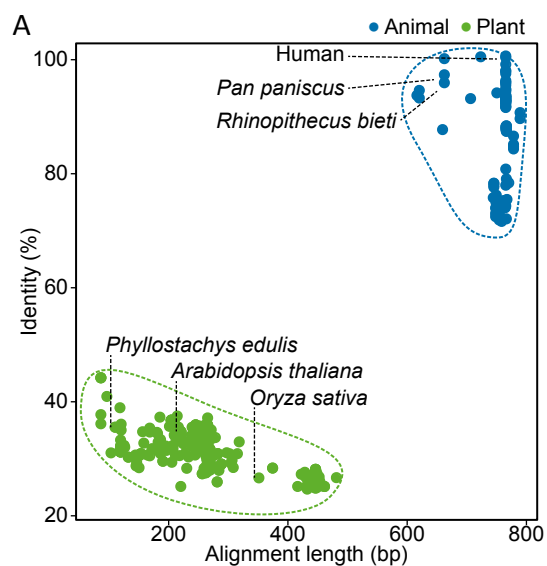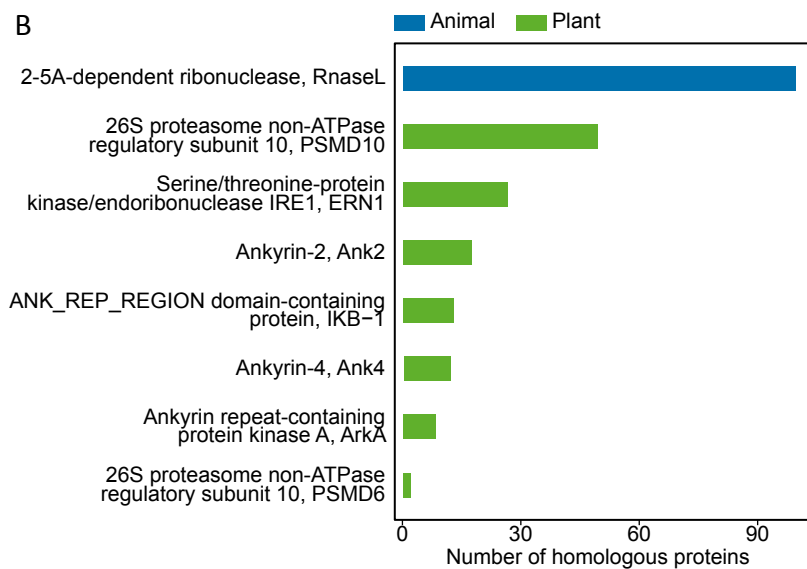

Supplement: Supplementary Figure S6 — Homology analysis of RNase L A. Multiple sequence alignment of RNase L in different species. B. The histogram plot shows the number of homologous proteins. [file mmc6.pdf]

A

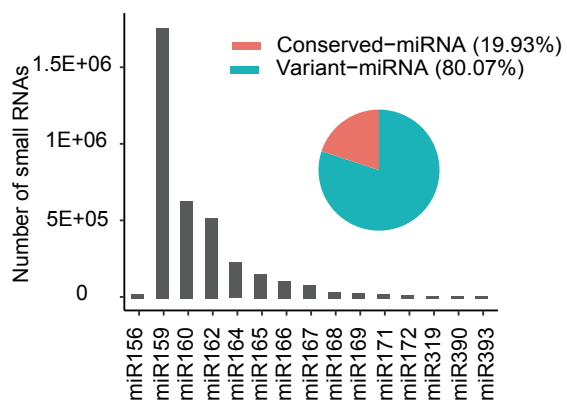

B

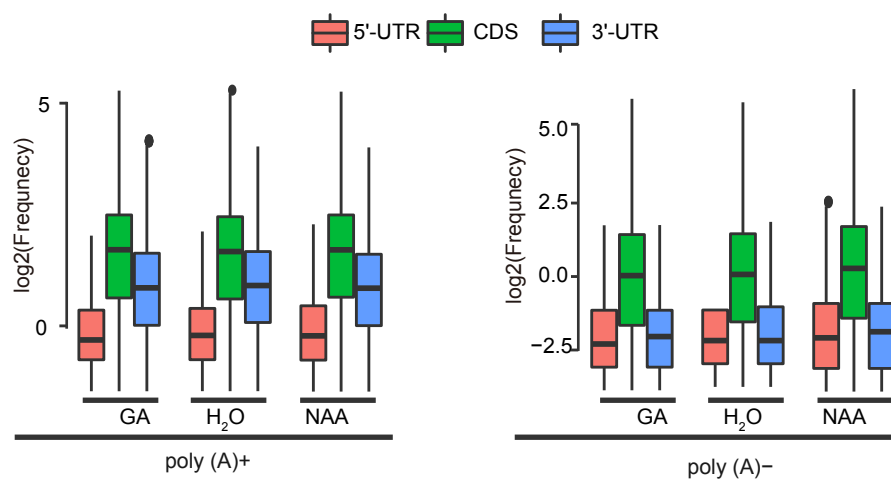

C

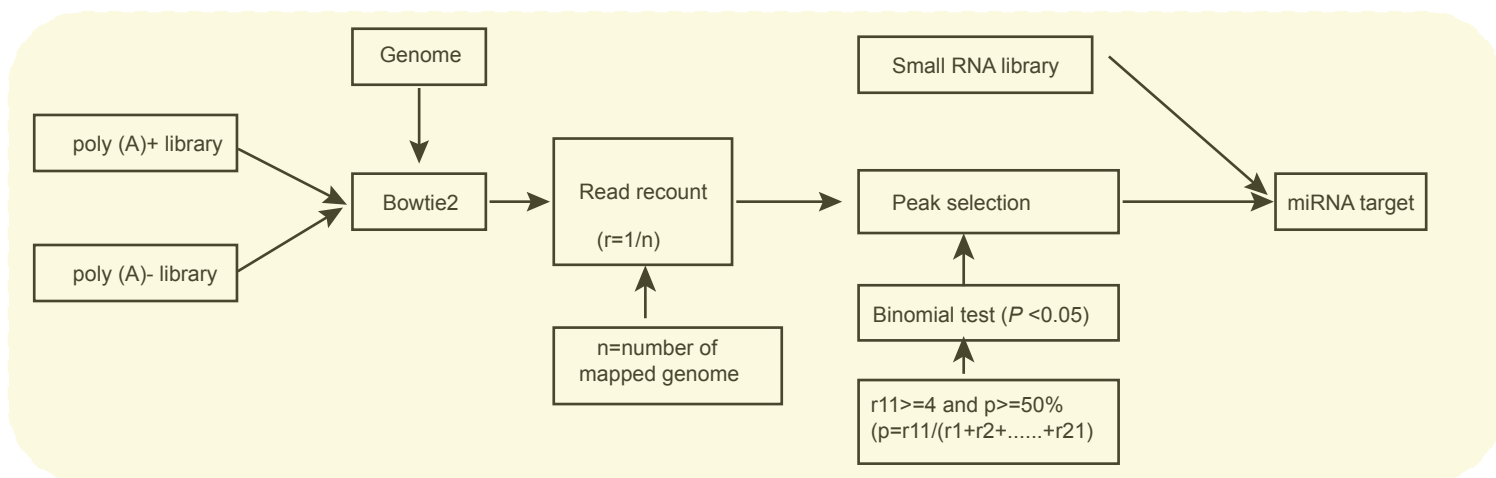

D

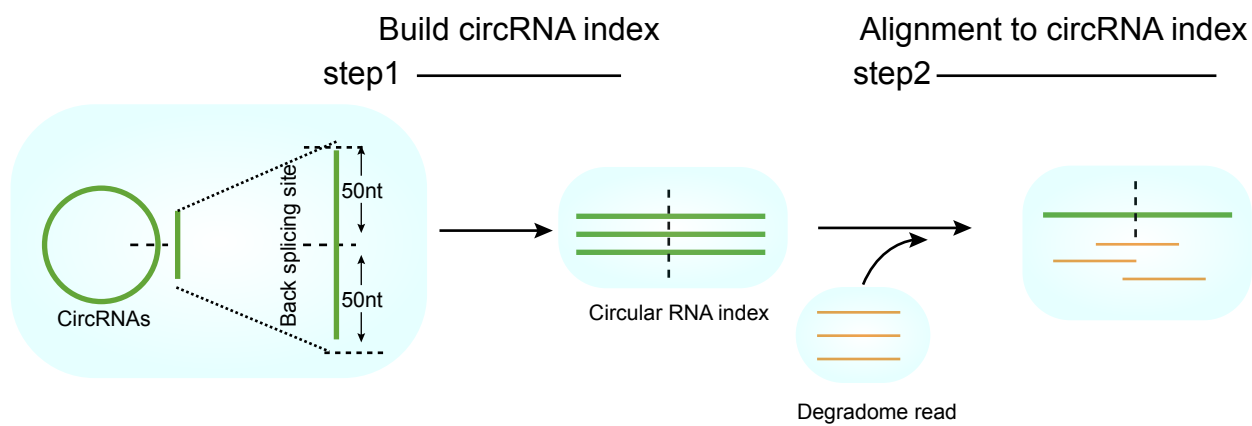

Supplement: Supplementary Figure S7 — Construction and analysis of degradome libraries A. The histogram plot shows the number of miRNAs and top 15 miRNA families. B. The boxplot shows the distribution of reads in the 5' UTR, CDSs, and 3' UTR from poly(A)+ and poly(A)− degradome libraries. C. A computational pipeline for identifying cleavage sites based on two types of degradome libraries. D. A computational pipeline for detecting decaying transcripts of circRNAs spanning back-splicing sites. [file mmc7.pdf]

A

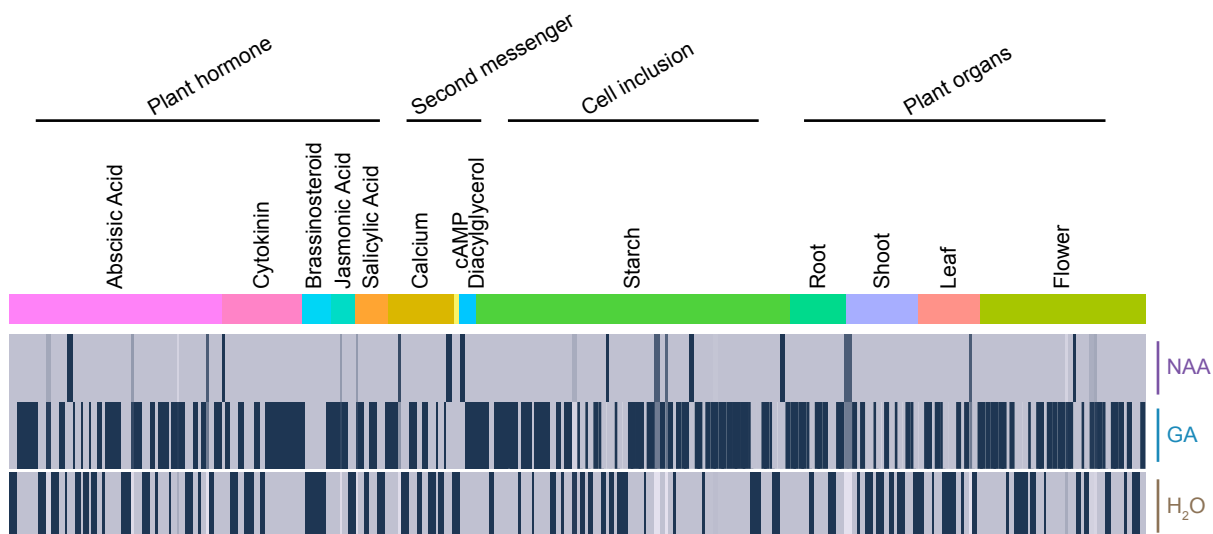

B

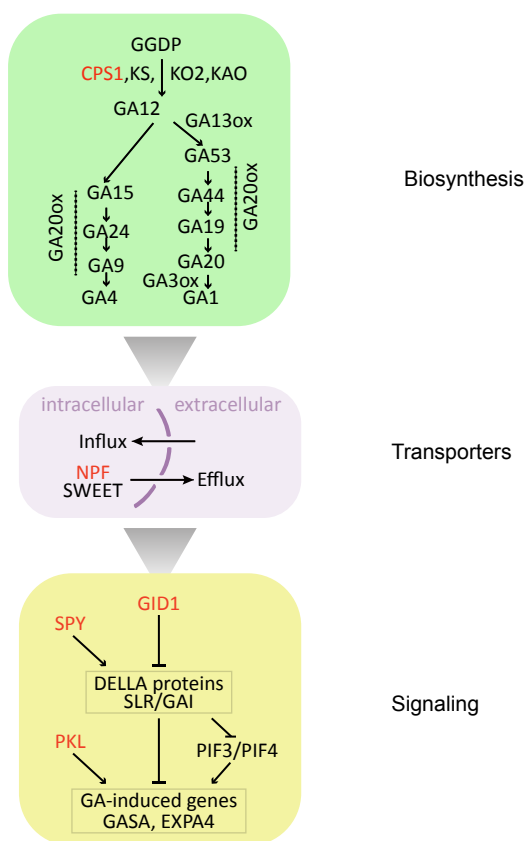

C

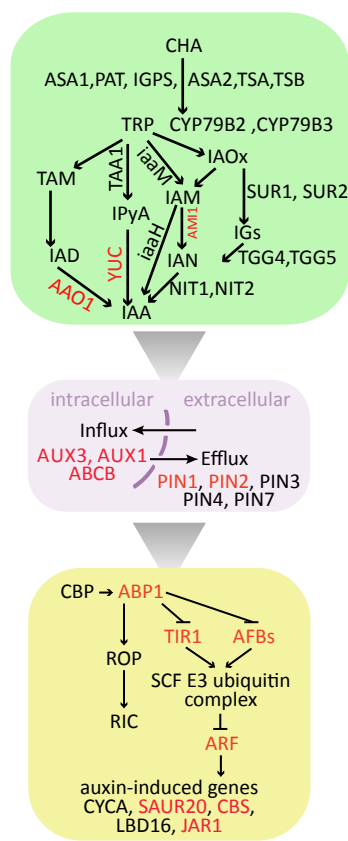

Supplement: Supplementary Figure S8 — Potential function of hormone-induced circRNAs A. Heatmap showing expression levels of circular transcripts related to the GO biological process terms plant hormones, second messenger, cell inclusion bodies, and plant organs. B. The events in the presence of gibberellin. C. The events in the presence of auxin. Red genes indicate host genes that generated circRNAs. The light-green box indicates biosynthesis, light purple indicates transport, and light yellow indicates signaling. Black arrows indicate positive effects, and black dashed lines indicate negative effects. [file mmc8.pdf]

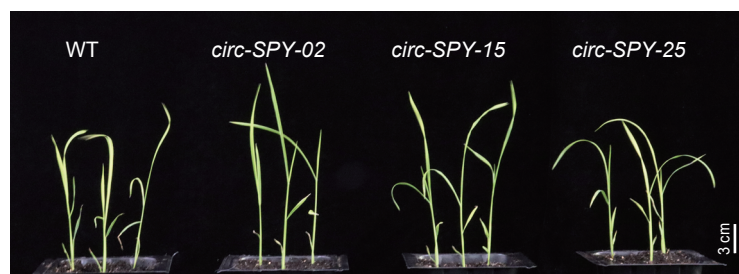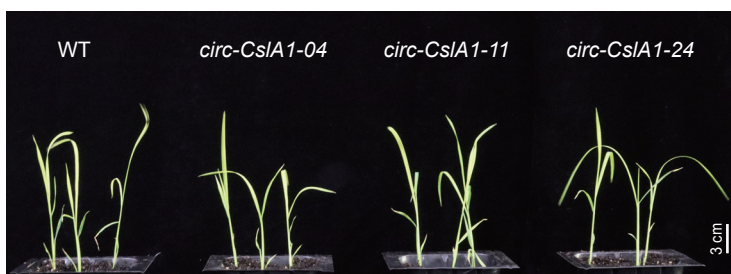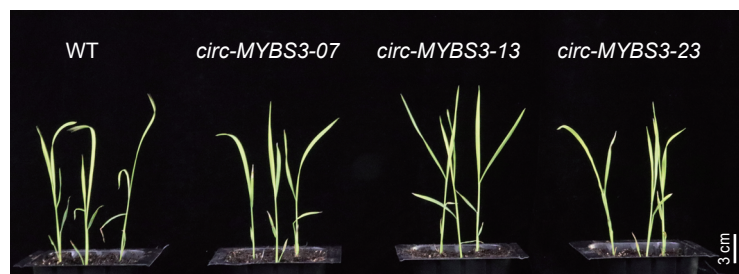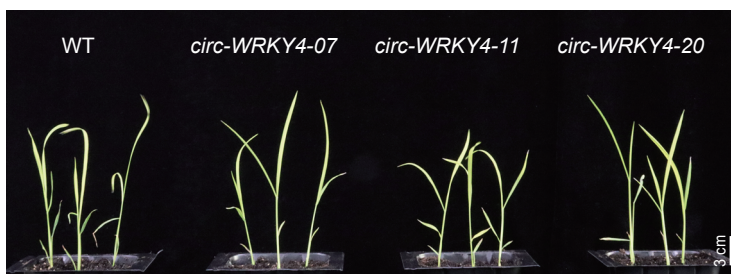

Supplement: Supplementary Figure S9 — The overexpression of six candidate circular RNAs (circ-SPY, circ-MYBS3, circ-WRKY4, circ-CslA1, circ-AGO1A, and circ-GID1) in rice. [file mmc9.pdf]
